# Amazonian Biomass Burning Enhances Tropical Andean Glaciers Melting

**DOI:** 10.1038/s41598-019-53284-1

**Published:** 2019-11-28

**Authors:** Newton de Magalhães, Heitor Evangelista, Thomas Condom, Antoine Rabatel, Patrick Ginot

**Affiliations:** 1 Laboratory of Geoprocessing, Institute of Geography, Rio de Janeiro State University, Rio de Janeiro, Brazil; 2 Geochemestry PHD program, Federal Fluminense University, Niteroi, Rio de Janeiro Brazil; 3 Laboratory of Radioecology and Global Change, Institute of Biology Roberto Alcantara Gomes, Rio de Janeiro State University, Rio de Janeiro, Brazil; 4 Univ. Grenoble Alpes, CNRS, IRD, Grenoble-INP, Institut des Géosciences de l’Environnement (IGE, UMR 5001), F-38000 Grenoble, France

**Keywords:** Cryospheric science, Environmental impact, Hydrology

## Abstract

The melting of tropical glaciers provides water resources to millions of people, involving social, ecological and economic demands. At present, these water reservoirs are threatened by the accelerating rates of mass loss associated with modern climate changes related to greenhouse gas emissions and ultimately land use/cover change. Until now, the effects of land use/cover change on the tropical Andean glaciers of South America through biomass burning activities have not been investigated. In this study, we quantitatively examine the hypothesis that regional land use/cover change is a contributor to the observed glacier mass loss, taking into account the role of Amazonian biomass burning. We demonstrated here, for the first time, that for tropical Andean glaciers, a massive contribution of black carbon emitted from biomass burning in the Amazon Basin does exist. This is favorable due to its positioning with respect to Amazon Basin fire hot spots and the predominant wind direction during the transition from the dry to wet seasons (Aug-Sep-Oct), when most fire events occur. We investigated changes in Bolivian Zongo Glacier albedo due to impurities on snow, including black carbon surface deposition and its potential for increasing annual glacier melting. We showed that the magnitude of the impact of Amazonian biomass burning depends on the dust content in snow. When high concentration of dust is present (e.g. 100 ppm of dust), the dust absorbs most of the radiation that otherwise would be absorbed by the BC. Our estimations point to a melting factor of 3.3 ± 0.8% for black carbon, and 5.0 ± 1.0% for black carbon in the presence of low dust content (e.g. 10 ppm of dust). For the 2010 hydrological year, we reported an increase in runoff corresponding to 4.5% of the annual discharge during the seasonal peak fire season, which is consistent with our predictions.

## Introduction

Large-scale deforestation and biomass burning in the Amazon Basin are direct consequences of economic practices and human occupation^1,2^. The agricultural frontier in the Amazon has advanced towards the natural rain forest since the 1970s, reaching dense forest domains where slash and burning occur^3^. This dynamic process is accompanied by biomass burning, which is the major source of particulate matter^4^, including aerosol black carbon (BC) (light-absorbing carbonaceous particles). BC is known to be a melting force once deposited onto ice sheets and high mountain glaciers, as described for Greenland^5–7^, western China^8^ and the Himalayas^9^. The deposition of high amounts of BC in these regions significantly reduces snow albedo and ultimately accelerates snow and ice melting. A survey of Andean tropical glaciers pointed to ice mass loss since the 1970’s^10–12^ which has increased attention on future catastrophic implications for the water resources of populations living in the western Andean region^13–15^. Intergovernmental panel on climate change (IPCC AR4 − 2007) models project an air temperature increase over the coming decades and that this rise will be amplified in the mid-to-lower troposphere at high altitudes in the Andean ridges of Ecuador, Peru, Bolivia, and northern Chile^16^. Models currently used to predict Andean glacier response to climate changes are primarily based on energy balance equations, which solely consider the impacts of solar and *in situ* meteorological conditions over glaciers^17^, neglecting the albedo reduction caused by the deposition of BC (i.e., the BC-albedo effect). An important point to consider is that biomass burning in South America largely occurs during the transition period between the austral dry and wet seasons (August to October) (Supplementary Material, Fig. 1a,b1); therefore, the wet removal of biomass burning smoke (including BC) in the Amazon Basin is low, and BC can be transported to distant regions located as far as the western Atlantic Ocean coast^18^ via the predominant wind (Supplementary Material, Fig. 2). Additionally, during this period, cloud cover is still low, and solar radiation is high over tropical Andean glaciers^19^, which makes the deposition of solid particles, such as dust and BC, of high relevance. Annually, the burning of forests and savannas in South America releases more than 800 Gg of BC^20–22^, which is higher than the estimations for fossil and biofuel emissions in Europe (470 Gg Yr^−1^ of BC)^20^. Brazil and Bolivia are the two countries most affected, accounting for 70% and 10% of fire event observations, respectively (Supplementary Material, Fig. 1b2). The number of fire events in Brazil and Bolivia were high from 2002 to 2004 due to high deforestation rates (Supplementary Material, Fig. 1b3). Since this period, Brazilian government increased investments in surveillance and adopted policies more conducive to forest conservation. As a result, deforestation and fire events decreased. Although conservation policies were efficient in reducing deforestation, the number of fire events was high in 2005, 2007 and 2010 because of El Niño events which caused extreme droughts in the Amazon. During drought condition fire can easily spread from the initial location. Since 2013, deforestation has slightly increased, which has raised the attention to the future of Amazon forest conservation and biomass burning emissions. Considering the proximity of the Amazon Basin to the tropical Andean glaciers and the dimensions of Amazonian biomass burning (Supplementary Material, Fig. 1c), an investigation of such impact is valuable.

In this study, we examined, for the first time, the hypothesis that biomass burning in the Amazon Basin can contribute to glacier mass loss in the tropical Andean mountains. Using a combination of observation and modeling approaches, we demonstrated that aerosols from biomass burning (including BC) can reach tropical Andean glaciers, leading to changes in the glacier surface energy balance and enhancing melting. We observed that a measurable effect on ice mass loss does exist considering such a mechanism. We believe that our results are of great relevance for the awareness of decision-making governs, people involve in land use politics and people affected by the use of melting waters from the Andes.

## Results

### Amazon basin smoke plumes in the central andes

**Figure 1:**
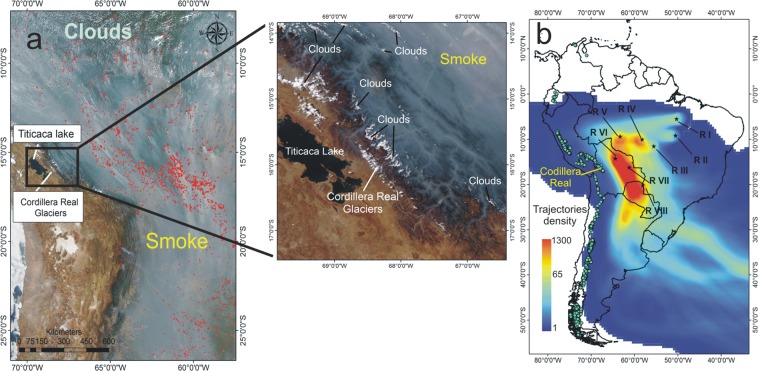
Smoke plume transport from the Amazon Basin to the Central Andes. (**a**) Formation of a mega-smoke plume resulting from the union of several biomass burning emissions (red dots represent the locations of fire events; clouds appear bright white and smoke appears in shades of grey). The images are daily MODIS true color band composition (1-red, 4-green, 3-blue) images from the Aqua satellite captured on 23 August 2010 (NASA Worldview application, link to the image: https://worldview.earthdata.nasa.gov). On that day, 148,946 fire events were registered in South America. (**b**) Trajectory density showing the average pattern of air mass trajectories during the biomass burning season from 2000–2016. Air mass trajectories at 6-hour time steps were analyzed each September (peak of the fire season in South America) from 2000 to 2016, resulting in a total of 2.040 trajectories. Trajectories were obtained using the HYSPLIT/NOAA model (https://www.ready.noaa.gov/HYSPLIT.php). Trajectory density (the number of trajectories over a 1.5 km radius) was calculated using ArcGIS^®^ 10.2 software. Light blue circles represent glaciers located in South America (data from the Randolph Glacier Inventory database^65^), and black stars (regions I, II, III, IV, V, VI, and VII) indicate the origins of the trajectories located within regions with a higher density of fire events.

Although fire ignition is caused by human activity, fire distribution intensity also depends on the severity of drought conditions that may take place due to ENSO warming events^3^ and anomaly warming conditions in the north tropical Atlantic Ocean^23^. Historically, it has been found that the number and intensity of fire events correlate with prolonged droughts^23–25^. During severe droughts in the Amazon Basin, smoke plumes may propagate through vast regions of South America, moving over the banks of the Andean Mountains, as previously detected from satellite observations in 2010^25^ (Fig. 1a). To detail this condition and to investigate where measurable impacts of fire events on ice cover do exist, we compiled all fire events occurring in the Amazon Basin from 2000 to 2016 (fire database was obtained from the Brazilian Institute of Space Research - INPE^26^). From this, we were able to identify major source regions of smoke plumes and to calculate the air mass forward trajectories using the HYSPLIT/NOAA model^27^ (Hybrid Single Particle Lagrangian Integrated Trajectory model) from high biomass burning regions. The model output shows the average pattern of aerosol transport from 2000–2016 (Fig. 1b) and clearly depicts that the Central Andes, mainly Bolivia and Peru, is a potential region for biomass burning smoke plume impacts as a result of the predominant east-to-west air circulation at the tropical-equatorial region of the Amazon Basin due to the Intertropical Convergence Zone (ITCZ)^28^. Such an impact can occur due to aerosol BC deposition on the glacier (among other biomass burning byproducts) and heat transport.

Among the glaciers located in the Central Andes, along the plume displacement, we chose the Zongo Glacier, located in Cordillera Real (−16.279°S; −68.142°W), Bolivia, as a study area (Supplementary Material, Fig. 3). Our choice of the Zongo Glacier was based on the availability of longer and continuous meteorological, surface mass balance and melting water discharge data for the glacier provided by the GLACIOCLIM program (https://glacioclim.osug.fr/) and the GREAT ICE program^29^.

One important point to observe is that any impacts of biomass burning on Andean ice can only be postulated if the convective rising of Amazon plumes can at least reach the altitudes of the mountain snowline. Over the Amazon Basin, previous LiDAR (Light Detection and Ranging) observations indicated that aerosol plumes during fire events can reach approximately 3.0–5.0 km in altitude^30^. Although Zongo Glacier extends from 4.9–6.0 km in altitude (the upper limit being beyond the smoke plumes), airflow striking the Andes Cordillera can be subjected to orographic effects that could lead the elevation of air masses to the altitude of the glacier. This mechanism was observed in South-Southeast Asia, where PM10 aerosols from biomass burning haze reached the upper troposphere due to orographic lifting over the Malaysian mountains^31^.

**Figure 2:**
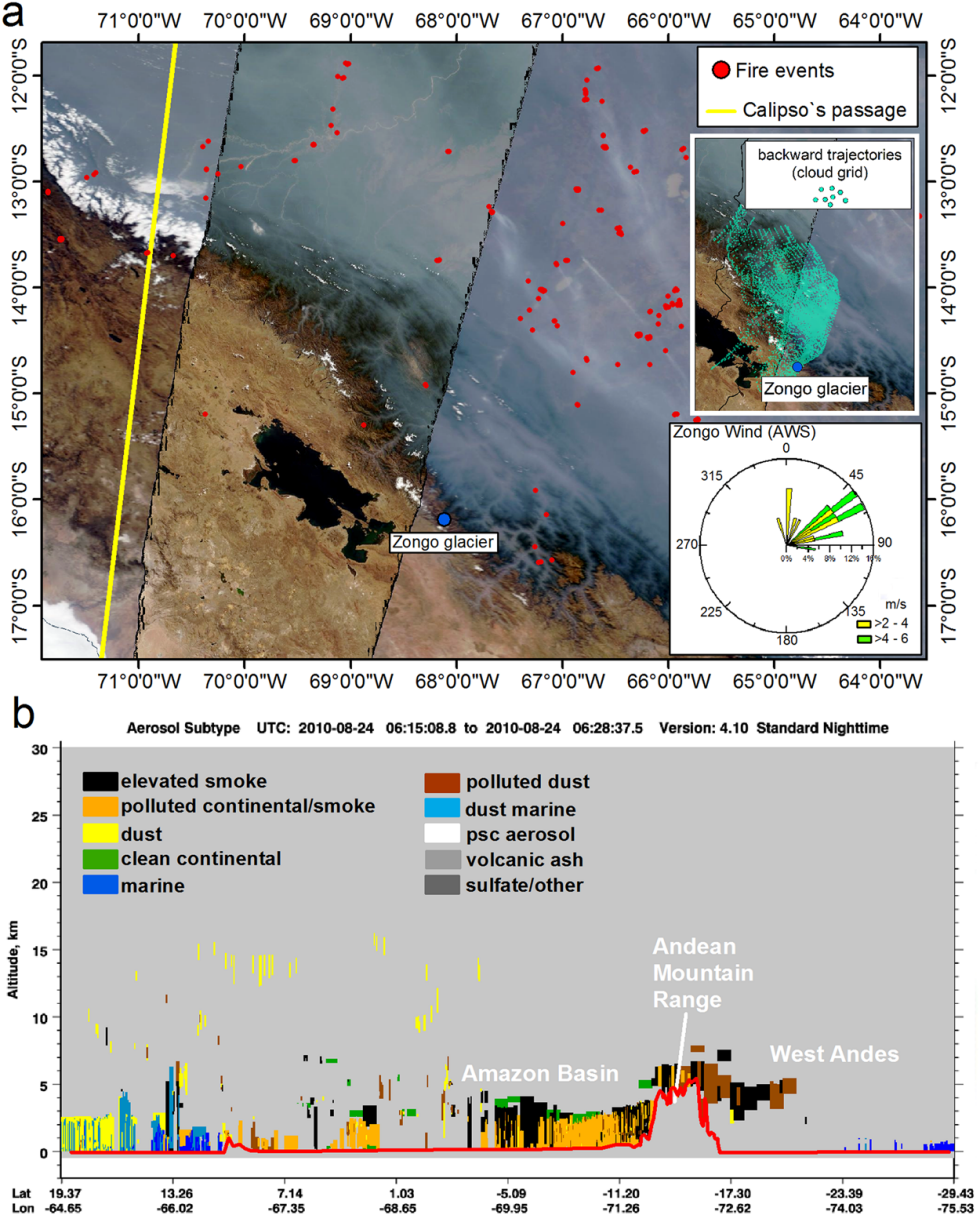
CALIPSO-CALIOP smoke transport analysis. (**a**) Daily MODIS true color band composition (1-red, 4-green, 3-blue) from the Aqua satellite captured on 08/24/2010 (NASA Worldview application, link to the images: https://worldview.earthdata.nasa.gov/08_24_2010). The Amazon Basin on the east side of the Andean Cordillera is completely covered by smoke from biomass burning. HYSPLIT backward trajectories show that wind masses that reach the Zongo Glacier come from the Amazon Basin (the wind direction at the Zongo Glacier corroborates with the HYSPLIT trajectories). (**b**) Vertical profile of aerosols over the Andean mountain range during the fire event on 08/24/2010 showing the presence of smoke above the Andean Cordillera.

Smoke transport analysis was conducted to prove that biomass burning emissions can reach the Bolivian glaciers. Herein, we detailed the vertical distribution and aerosol types from two transects from the Amazon Basin to the Central Andean highlands encompassing the region of the Zongo Glacier using remotely sensed LiDAR observations of aerosols from CALIPSO (Cloud-Aerosol Lidar and Infrared Pathfinder Satellite Observations) over the 2010 fire season (24 August 2010 and 11 September 2010). On August 24, 2010, the presence of smoke plumes over the Bolivian Amazon was clearly identified in cloud-free regions (Fig. 2a). During this period, numerous fire events were concentrated near the eastern side of the Andean Mountains within the Bolivian and Peruvian portions of the Amazon Basin. Meteorological data recorded at the Zongo Glacier indicated that northeastern and eastern winds were predominant (Fig. 2a). Such wind directions favor smoke plume displacement from the Amazon Basin towards the Andean glaciers. The CALIOP sensor (onboard CALIPSO) recorded an aerosol plume over the Andean Mountains containing the same composition as the aerosol plume located over the Amazon Basin (Fig. 2b). The plume contained mainly dust and smoke pollution. This finding confirms that smoke plumes from Amazonian biomass burning are capable of reaching the top of the Andean Mountains between altitudes of 5.0 and 6.0 km. On September 11, 2010, the plume over the Central Andes contained polluted continental/smoke, dust, and polluted dust. The polluted continental/smoke can be observed from the Amazon Basin to the top of the mountain range. No aerosol plume is observed on the west side of the Andes, whereas the polluted dust and dust observed over the Andean mountains should be caused by local production driven by wind (supplementary material, Fig. 4).

### Estimating black carbon concentrations at Zongo Glacier

Since the start of the MODIS satellite fire monitoring program in 2002, modeled emissions of BC data available at the global fire emission database (GFEDBv4) noted the 2007 and 2010 fire seasons as most critical for the Amazon Basin (Supplementary Material, Fig. 5a,b). Therefore, to assess the potential maximum impact of BC on the Zongo Glacier, we focused on these episodes. From the above, we modeled continental BC aerosol emissions, atmospheric concentration and deposition from the HYSPLIT/NOAA model. The BC emissions used in our model were from a global fire emission database, which uses a compilation of emission factors retrieved from experiments that include the measurements of BC in the Brazilian Amazon forest during biomass burning events^32^. From our results, atmospheric BC concentrations values due to Amazonian biomass burning emissions ranged from 0 to 5.0 × 10^−3^ mg m^−3^ in the Central Andes (Supplementary Material, Figs 6 and 7), and total BC depositions on the Zongo Glacier were estimated as 0.88 mg m^−2^ and 1.17 mg m^−2^ during the fire seasons of 2007 and 2010, respectively (Supplementary Material, Fig. 8). These predictions are in agreement with the measurements of BC at the Chacaltaya station (5 km from Zongo Glacier), where atmospheric concentrations of black carbon ranging from 0.2 to 1.5 × 10^−3^ mg m^−3^ were reported (database from 2012–2014)^33^ and with other global aerosol models that estimate BC deposition values between 0.5–2.0 mg m^−2^ for the Andes^34^.

Modeled BC deposition and measured snowfall at the glacier surface were used to calculate BC concentrations in snow at the Zongo Glacier (details are presented in the methods section). Liquid precipitation over the glacier is rare and, therefore, neglected in the model. Our estimated BC concentrations in snow were 41.1 ppb (parts per billion) for September 2007, 73.4 ppb for September 2010, and 29.2 ppb at the end of the fire season in October 2010. The lowest concentration (29.2 ppb of BC) in October 2010 was attributed to the high precipitation over the glacier during that period, which diluted the concentration accompanied by a decrease in fire events in the Amazon Basin. The high BC concentrations estimated for September each year were related to the peak in fire events in South America combined with associated low precipitation rates in the Andes. A comparison of our modeled concentrations with Illimani ice core data shows consistency. The Illimani Glacier is located approximately 55 km southeast of the Zongo Glacier, and due to its geographic proximity, it has a similar influence on the transport of particles from the Amazon Basin (from our model). The BC record from the Illimani ice core displays a strong seasonality, with low values during the wet season and high values during the Amazon fire season (Supplementary Material, Fig. 9), which is consistent with the observed transport of biomass-burning aerosols from the Amazon Basin. BC measurements performed on the Illimani ice core displayed a peak of 58.3 ppb of BC for 2007, which was slightly higher than the concentration of 41.1 ppb that we modeled for the same period for the Zongo Glacier (Supplementary Material, Fig. 9). In fact, our modeled values solely considered biomass burning contributions, whereas emissions from fossil fuels from nearby urban sites likely contributed, to some extent, to the total BC deposition^35^. Furthermore, post depositional processes must be important and may explain why our BC concentration values in snow were below the measured values in the Illimani core. For investigating major causes of BC interanual variability we examined spatial/temporal correlation between the Illimani BC record and the aerosol index over the South America from 1994 to 2009 (Supplementary Material, Fig. 10). We found significant positive correlation (p < 0.05) between Illimani BC and aerosol index for areas in the Amazon Basin located East of the Illimani Glacier, where extensive fires occur during the fire season. On the other hand, we did not find significant correlation between Illimani BC and aerosol index at La paz/ El alto region. Our results indicate that Amazon Basin is the main source region of the BC deposited at Illimani.

### Reduced snow albedo and enhanced energy flux due to black carbon and dust at Zongo Glacier

Very few studies have investigated the impurity content in Andean low-latitude glaciers. Measured dust concentrations ranging from 1 to 9 ppm were reported for the Quelccaya Glacier, Peru (5670 m asl)^36^. During the wet season, impurity contents ranging from 10 ppm in fresh snow to 100 ppm in 1-week-old snow^37^ were observed on the Zongo Glacier. Here, we investigated the snow albedo reduction and the consequent enhanced energy flux at the Zongo Glacier due to BC only and BC in the presence of previously reported dust concentrations (10 ppm and 100 ppm of dust). Considering solely the BC concentrations in snow, we performed estimations using the SNICAR snow albedo simulation^38^, with an albedo reduction ranging from 1.8 to 7.2% (Supplementary Material, Fig. 11). Again, our results are in agreement with radiative transfer calculations, in which BC concentrations in snow on the order of 10–100 ppb correspond to decreases in albedo by 1–8%^8,39,40^. Considering the albedo reduction due to BC in the presence of 10 ppm and 100 ppm of dust, we estimated reductions ranging from 3.8 to 9.6% and 11.5 to 20.2%, respectively (Supplementary Material, Fig. 11). These results are in agreement with the observations that visible snow albedo during the dry season on the Zongo Glacier can be reduced by 10–20% due to atmospheric aerosols contained in snow^37^.

The BC contribution to energy flux on the snow/ice surface for the period from 8 September to 31 October 2010 (corresponding to the Amazon fire season) was assessed (Supplementary Material, Fig. 12a). The BC contribution to the energy flux reached a maximum during the peak of the Amazon Basin fire season in mid-September, 10.7 ± 3.5 W m^−2^. Following this period, the energy flux contribution decreased to values between 4.7 ± 1.6 and 1.3 ± 0.4 W m^−2^ by the end of October since in this period, BC deposition rates were reduced, while precipitation rates and cloud cover tended to increase (consequently lowering incident solar radiation at the glacier’s surface and diluting the concentration in the snow/ice surface). The sum of the variables radiative balance (R), sensible heat flux (H), latent heat flux (LE) and forcing due to BC corresponds to the total energy flux at the glacier’s surface (Supplementary Material, Fig. 12a). Radiative balance (R) is the sum of the net incoming and outgoing shortwave and longwave radiation. It is the main source of energy for the glacier’s surface and may significantly vary over time (days). Following BC deposition, R presents higher values during September and lower values during October. Most of the time, the sensible heat flux was positive, whereas the latent heat flux remained negative. The sum between the radiative balance and sensible and latent heat fluxes was positive all of the time, even when the latent heat flux was high. The result indicates that although a portion of energy was consumed by sublimation, sufficient energy for generating snow/ice melting conditions during the fire season was present. Based on the above, BC is evidently an effective “melting parameter” since it positively contributes to an increase in the radiative balance due to the albedo effect, thus intensifying melting. The increase in energy flux used to melt snow/ice (QM) due to BC was 4.2 ± 1.4% in September and 3.6 ± 1.2% in October 2010 (Supplementary Material, Fig. 12b).

### Enhanced mass loss due to black carbon and dust at Zongo Glacier

A snow/ice surface mass balance model was applied for Zongo Glacier based on a linear regression between QM at the surface and the *in situ* annual mass balance measured at the glacier between 2005 and 2011. Simulations were made at an elevation range of 5.100–5.200 m a.s.l., which was where the automatic weather station was operated and where the surface mass balance measurements were conducted. The linear regression between the surface mass balance and the energy balance show statistical significance (statistical significances were based upon a Student t test, p < 0.001, n = 6, r² = 0.96). The coefficient of determination (R²) between the measured and modeled surface mass balances was 0.96, indicating the ability of our model to accurately reproduce the observed surface mass balance (Supplementary Material, Fig. 13).

**Figure 3:**
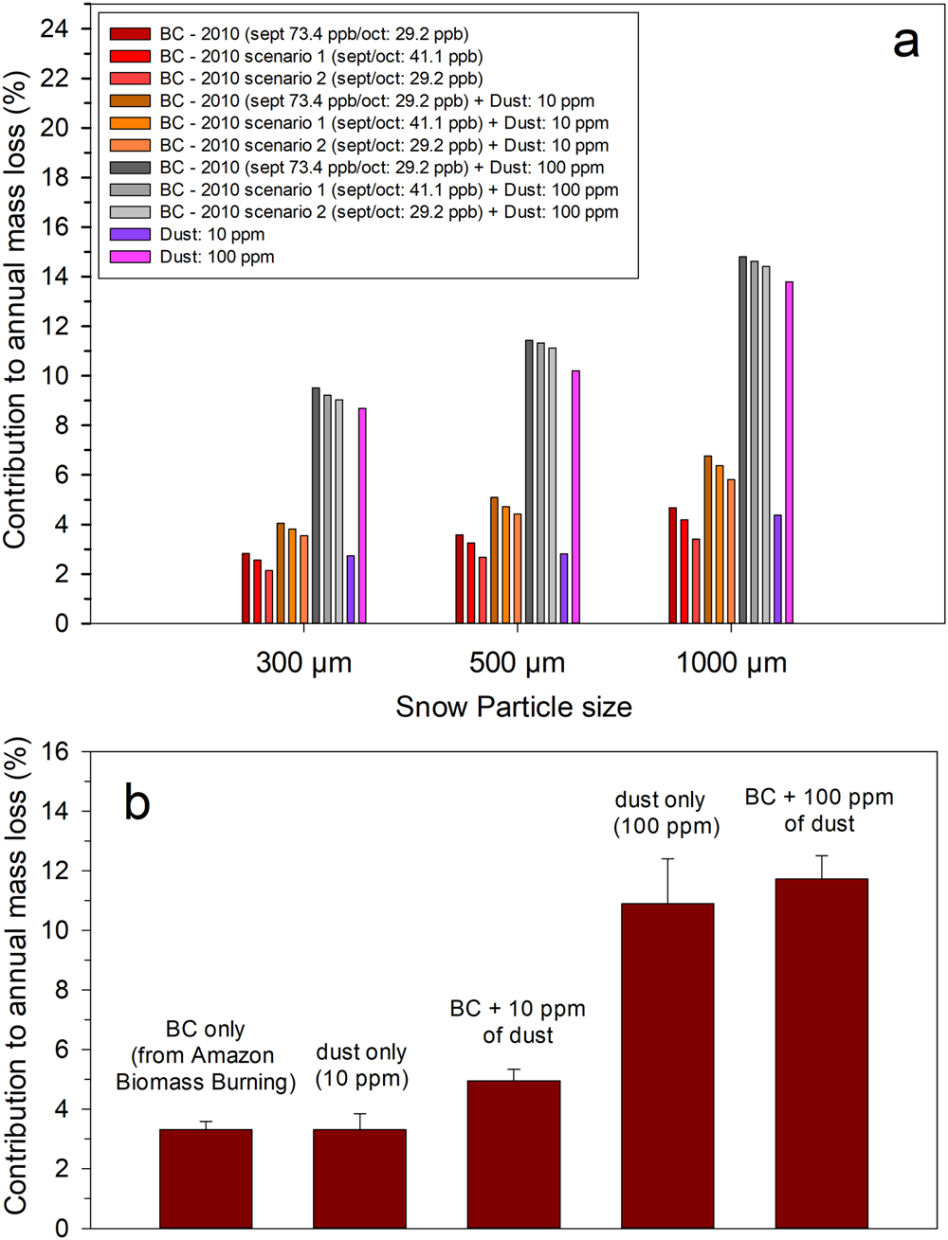
Contribution of aerosol particles in snow to mass loss at Zongo Glacier. (**a**) Potential annual snow/ice mass loss due to BC and dust for different scenarios. (**b**) Bar plot showing the average potential contributions of BC and dust to annual mass loss at the Zongo Glacier.

Mass loss due to BC was estimated for 2010 by considering varying BC concentrations for September and October 2010. Additionally, two other estimations were conducted, taking into account two different conditions. The first was based on constant BC concentrations of 41.1 ppb (representing the BC concentration modeled for September 2007); the second was based on constant BC concentrations of 29.2 ppb (representing the BC concentration modeled to the end of October 2010). For each condition, we also considered the presence of dust (10 ppm and 100 ppm) and different snow grain sizes (300 µm, 500 µm and 1000 µm) (Fig. 3a). Based on these estimations, BC from Amazonian biomass burning has the potential to increase annual melting by 3.3 ± 0.8%, whereas dust alone can increase annual melting by 3.2 ± 0.9%, when dust content is low (10 ppm of dust) and by 10.9 ± 2.6%, when dust content is high (100 ppm of dust) (Fig. 3b). In the combined presence of BC and dust, annual melting can increase from 5.0 ± 1.0% to 11.7 ± 2.3% (Fig. 3b). For comparison, this value is comparable to that for glaciers in Kyrgyzstan (central Asia), which have been documented to have an increase in snow melt rates of 6.3% due to the BC-albedo effect^39^.

### Comparing biomass burning “forcing” with water discharge at Zongo glacier

**Figure 4:**
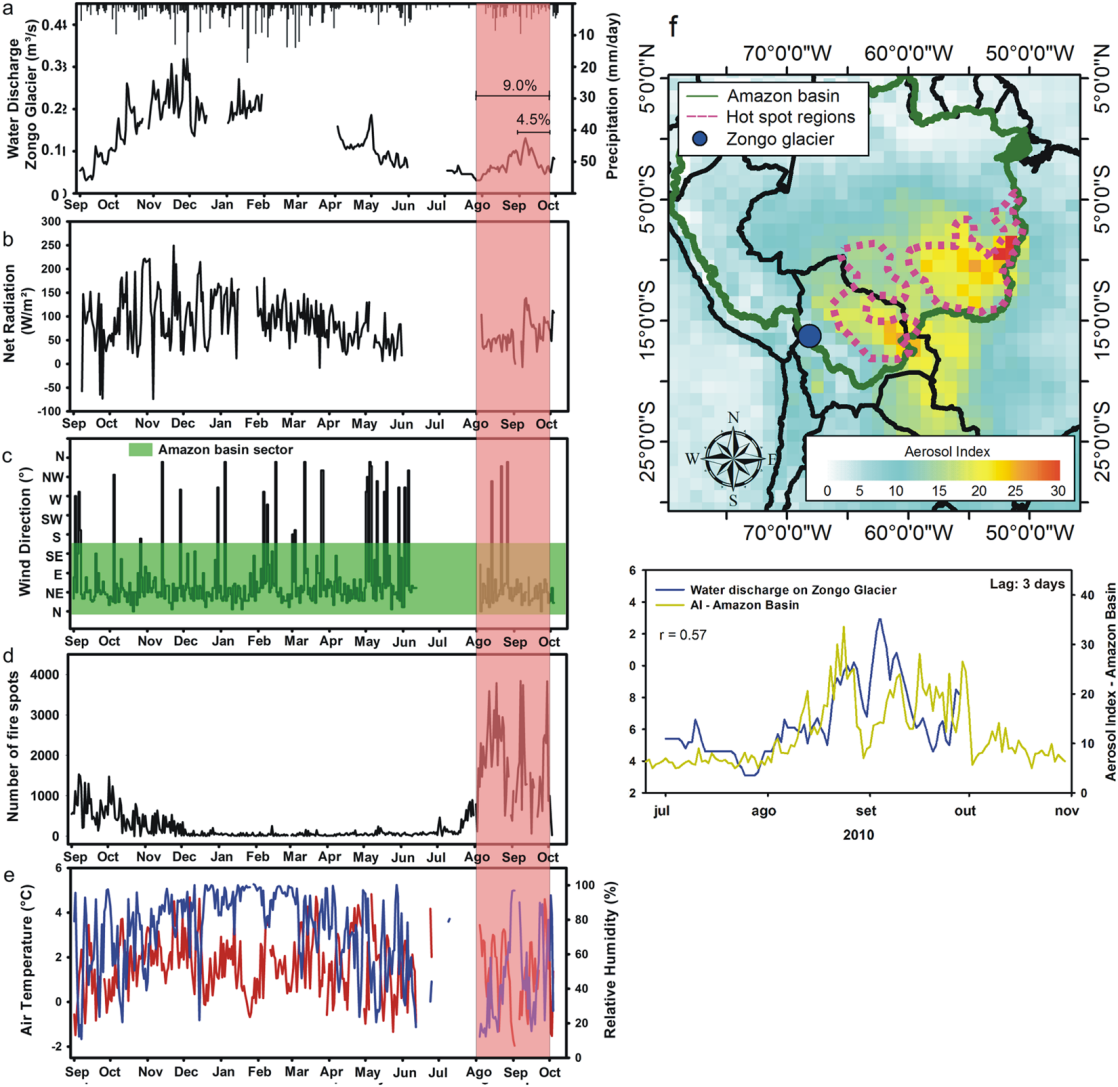
Hydrological and meteorological conditions of Zongo Glacier during the 2010 fire season. (**a**) Daily water discharge at Zongo Glacier during the 2009/2010 hydrological year and precipitation over the glacier (AWS data). (**b**) Daily fire outbreaks within the Amazon Basin (the INPE fire database). (**c**) The wind direction (AWS data). (**d**) Incident solar radiation (AWS data). (**e**) Air temperature (red) and humidity (blue) of the Zongo Glacier (AWS data). (**f**) Monthly aerosol index map for September 2010. The green line represents the geographical limit of the Amazon Basin, the blue dot represents the location of the Zongo Glacier, and the pink dashed line represents the hot spot regions for fire events used for the analysis of AI time series. (**g**) Water discharge of Zongo Glacier and the AI within the Amazon Basin during the peak of the 2010 fire season.

A database of *in situ* interannual water discharge was used for direct comparison with the estimated mass balance. Water discharge behaved similarly to precipitation, displaying a major increase between October and February in 2009/2010 and following radiation flux seasonal variability for the tropics (Fig. 4a,b). A steady decrease in discharge was observed from February to August 2010. During the dry season (May-July), net radiation was slightly lower, and almost no precipitation was observed on the glacier (Fig. 4a,b). The dry period was marked by western/northwestern winds (Fig. 4c), which prevent humidity flow from the Amazon Basin to the eastern Andes^41,42^. A synoptic analysis indicated that days with easterly winds during the dry period were uncommon, occurring approximately 5% of the time^41^; consequently, low humidity and western winds favored sublimation at the glacier’s surface, consuming much of the available energy and resulting in low melting^41,43^. From August onward, the predominant wind direction changed from western/northwestern to eastern/northeastern (Fig. 4c). This change is associated with the weakening and southward migration of the subtropical jet that occurs due to the southward migration of the ITCZ in conjunction with the establishment of the Bolivian high^42^. This change in the predominant wind direction results in the prevalence of a weak easterly wind in the middle and upper troposphere within the Central Andes Altiplano. Such a change occurs during the fire season and favors smoke transport to the glacier (Fig. 4c,d). One may observe from Fig. 4a that a secondary peak in water discharge does exist from the beginning of August to the end of September, occurring outside the rainy season, comprising approximately 9% of the total annual discharge. This secondary peak in water discharge began in August and extended until the end of September, coinciding with the fire season of the Amazon Basin (Fig. 4a,d) and following the increase in aerosol emissions from biomass burning (Fig. 4f,g). For September 2010, water discharge corresponded to 4.5% of the annual discharge, which was approximately the same magnitude as expected for glacier runoff due to the impact of BC and mineral dust for the same period (3.3 ± 0.8% for black carbon only; 5.0 ± 1.0% for black carbon in the presence of 10 ppm of dust). Net radiation flux and temperature were maintained nearly the seasonal variability over this period (Fig. 4b,e). Additionally, precipitation was low over the glacier, indicating that the increase in water discharge most likely resulted from an increase in snow/ice melting at the glacier’s surface.

We estimated by the HYSPLIT/NOAA model that three days are required for smoke plume displacement from the emission source to reach the glacier (Supplementary Fig. 14). In fact, peak water discharge from 5 August 2010 to 18 September 2010 displayed a statistically significant correlation with the three-day lag, in the daily MODIS aerosol index for the Amazon Basin, r = 0.57 (n = 45; p < 0.05) (Supplementary Fig. 14). Among the predictor variables (*in situ* meteorological parameters and aerosol index of the Amazon Basin), the aerosol index and radiation flux (incident shortwave radiation and net longwave radiation) were statistically significant predictors of water discharge, explaining 70% of the variance (t-tests for partial coefficients, p < 0.01; for the full model, F_266_ = 27.74, p < 0.001; r^2^ = 0.70) (Supplementary Fig. 15).

## Discussion

Based on observations and modeling, we found that smoke plumes from the Amazon Basin can overcome the orographic barrier and reach the tropical Andean glaciers. These glaciers, which are located within the outer tropical Andes, are subject to the greatest impact of BC emissions from biomass burning within the Amazon Basin. During the peak fire season, the radiative balance is the main source of energy for the surface of Zongo Glacier. This turns the content of impurities at glacier surface a relevant melting forcing since deposited BC and dust increases the shortwave radiative balance due to the albedo effect, which can intensify annual melting from 5.0 ± 1.0% to 11.7 ± 2.0%. Our results reveal that the contribution of BC from biomass burning to surface melting do not differ in magnitude to the contribution of dust aerosol, when dust content is low (e.g. 10 ppm of dust). In this case, BC and dust alone can increase annual melting by 3.3 ± 0.8% and 3.2 ± 0.9%, respectively. Both combined can increase annual melting by 5.0 ± 1.0%. These increases in annual melting due to the combination of BC and dust showed statistically significant difference from the effect of BC and dust alone (t-test, p < 0.05). However, in the presence of high content of dust (100 ppm of dust) the efficacy of the biomass burning to cause melting is lower. Dust alone (100 ppm of dust) can increase annual melting by 10.9 ± 2.6%, whereas the combined effect of dust and BC may result in an annual increase of 11.7 ± 2.0%. In this case, the increase in annual melting due to the combination of BC and dust did not show statistically significant difference from the dust effect alone. This occusr because when high concentration of dust is present, the dust absorbs most of the radiation that otherwise would be absorbed by the BC. Thus, the magnitude of the impact of Amazonian biomass burning depends on the dust content in snow. Unfortunately, there are no measurements available to document the seasonal variability of the dust content on the Zongo Glacier. Though, previously spectral reflectance measurements on fresh snow during the dry season indicates a low dust content (<100 ppm of dust)^37^. Furthermore, we found a statistically significant correlation between peak water discharge at the Zongo Glacier and aerosol index for the Amazon Basin during the 2010 fire season (one of most critical fire seasons registered). Thus, we believe that such an impact even low, does exist, particularly for years with a high incidence of biomass burning within the Amazon Basin, and should be accounted for in future considerations on Andean water resources. Future projections of the Intergovernmental Panel on Climate Change (IPCC), which are equally important, point to a drier eastern Amazon climatology; this is a favorable scenario for increasing biomass burning risk^44^. In addition, in an Amazon Basin deforestation scenario, almost all models show changes in the South American hydrological cycle and monsoons with important reductions in precipitation and increases in aridity^45,46^. Not less important is the economic pressure related to the global food demand, which may lead to a progressive expansion of the Brazilian agriculture domains, resulting in an enhanced projection of BC and CO_2_ emissions, as in past decades. Biomass burning over southwestern Amazonia (which comprises the Brazilian, Peruvian and Bolivian Amazon) cannot be considered a regional issue to be faced but instead has social implications at the continental scale, making the use of water by several Andean communities a vulnerability.

## Methods

### Determination of BC emission source regions and the calculation of trajectory density

We used the fire database from the Brazilian Institute for Space Research (INPE)^26^ (which uses the collection 6 MODIS active fire detection algorithm^47^) to locate regions with the highest density of fire events spanning the period from 2000 to 2016. To determine fire event densities, we applied two spatial analyses: the kernel density analysis^48^ and the spatial statistic GI*^49^ using ArcGIS 10.2 software. The results of the two analyses supported one another, that is, regions with the highest density of fire events defined by the kernel analysis coincided with regions delimited by the GI* analysis as “hot spots”. The highest density of fire events occurred in Brazil, in the region known as the deforestation arc, and in the southwestern Amazon basin, which comprises the Bolivian Amazon. Based on these results, we defined seven regions as the sources of fire emissions to be used for the modeling of air mass trajectories in the HYSPLIT model (Supplementary Methods, Fig. 1).

### Observations of smoke plumes over the Andes

To verify whether smoke plumes from wildfires within the Amazon Basin can reach the top of the Andes Mountain range, we analyzed two paths of the CALIOP (Cloud-Aerosol Lidar with Orthogonal Polarization) sensor onboard the CALIPSO (Cloud- Aerosol Lidar Infrared Pathfinder Satellite Observations) satellite. The instrument records information regarding the vertical distribution of aerosols within the atmosphere, supporting the identification of air masses and the characterization of different types of aerosols^50,51^. CALIPSO’s algorithm for aerosol type classification uses the optical, geophysical, and temporal characteristics of the aerosol layer as decision points to navigate a flow chart that ultimately selects the most likely aerosol model for each aerosol layer^52^. The aerosol types, including “polluted continental”, “biomass burning”, “desert dust”, and “polluted dust”, are derived from a comprehensive cluster analysis applied to AERONET data gathered from numerous sites around the globe. “Clean continental” and “marine” sources are synthesized using measurements of long-range continental transport and sea salt acquired using a backscatter nephalometer^52^. Products from the CALIOP sensor were obtained from the NASA website: https://www-calipso.larc.nasa.gov/search/index.php. The dates analyzed were 24 August 2010 and 11 September 2010. These dates were chosen by taking into account the availability of data during extreme fire events and the presence of smoke plumes near the Andes Mountain range.

### Modeling the emission, transport, deposition, and impact of BC on the Zongo Glacier

Our work presents a methodological framework, that is, a set of steps and methods, for modeling the impact of BC aerosol on the Glacier. The methodological framework is presented in the Supplementary Methods (Supplementary Methods, Fig. 2). A brief explanation of the framework is as follows:

1. The first stage of modeling includes determining the emission regions to be inserted into the HYSPLIT model. For this step, the locations of fire events were determined, and the spatial density statistics were performed.
2. For each delimited emission source, total BC emissions were calculated using BC emission data produced by the Global Fire Emission Database - GFEDv4^53^ (available at http://www.globalfiredata.org/). We constructed a time series of BC emissions for each region from 2000 to 2011. This step was important in determining the most critical years of BC emissions and the emission rates to be used within the HYSPLIT model. Since the data are available monthly and since the HYSPLIT model takes into account the emission rate per hour, we assumed that the BC emissions were constant each month. In other words, we assumed that the total monthly emissions were equal to the number of hours in each month. The years 2007 and 2010 displayed higher BC emissions and numbers of fire events. Therefore, they were chosen for the modeling procedure.
3. Source emission regions and emission data were used as input data for the HYSPLIT model. We also used NCEP/NCAR reanalysis data^54^ as input meteorological data. The meteorological data employed were as follows: temperature, u-velocity, v-velocity, pressure vertical velocity, geopotential height, relative humidity (upper air data), 2 m temperature, 10 m u- and v- velocity components and precipitation (surface data), and the surface geopotential field (terrain height). Other parameters utilized included particle size = 0.32 μm (mode of particles during the fire season in the Amazon Basin^55^); particle density = 1.8 (g/cc)^56^; and an almost spherical particle shape (these parameters are used to compute particle settling velocities using Stokes law). BC from wildfires was assumed to be 60% soluble, meaning that in the presence of a cloud comprising 60% aerosols was captured as water in the cloud^32^. For high mountains regions, in-cloud mass scavenging efficiencies (MSEs) of BC were reported to be approximately 0.6^57,58^. In the HYSPLIT model, wet removal was defined as a scavenging coefficient expressed directly as a rate constant and modified by the precipitation rate (P in mm/h). The rate constant (β) was given by βwet = 0.6 P^0.79^. The residence time of BC in the atmosphere was assumed to be seven days. Similar parameters are generally employed in global BC transport and deposition models^32^^.^ Model runs were conducted to simulate BC transport and deposition for the fire season (August, September and October) during 2007 and 2010. Table 1 summarize the model parametrizations.

**Table 1:** Summary of the aerosol model characteristics.

Aerosol model	Meteorological data	BC emission data	BC lifetime in days^32^	Particle size^55^	BC density in g cm^−^³ ^56^	Wet removal^32,57,58^	Particle settling velocity	References for aerosol parametrizations
HYSPLIT	NCEP/NCAR reanalysis	GFED	7.0	0.32 µm	1.8	0.6	Calculated from particles characteristics	Koch *et al*.^34^ Martin *et al*.^55^ Zhang *et al*.^56^ Feng.^57^ Yang *et al*.
4. Simulated BC deposition and precipitation data from an automatic weather station located on the glacier were used to calculate the BC concentration at the glacier’s surface. The calculation was performed as follows:
  1 $${\rm{BC}}\,{\rm{concentration}}=\frac{{\rm{ng}}({\rm{bc}})}{{{\rm{m}}}^{2}\ast {\rm{t}}}.\frac{1}{{\rm{\rho }}({\rm{snow}})}.\frac{1}{p}$$
  The first term of the equation is the simulated BC deposition (ng. m^−2^.t^−1^), where ρ (snow) is the snowpack density (250 kg.m^−3^ at the Zongo Glacier for the end of the wet season^59^) and p is snow precipitation on the glacier. We assumed that snow was consistently present on the surface and that post-depositional processes did not occur. In other words, we assumed that the concentration of BC did not increase due to snow melting.
5. To simulate albedo changes due to BC, calculated BC concentrations were used as input data within the SNICAR model (the Snow, Ice, and Aerosol Radiation model). The SNICAR model takes into consideration the concentration of impurities within the snow (BC and dust), the snow particle size, some glacier characteristics, and the incident radiation flow^6,60^. Incorporated parameters included (a) a mean solar zenith angle of 22.55°, which was calculated for the fire season (Aug-Sept-Oct) using solar position calculations developed by the Solar Radiation Monitoring Laboratory at the University of Oregon (http://solardat.uoregon.edu/SolarPositionCalculator.html); (b) snow grain effective radii of 0.3, 0.5 and 1 mm based on measurements performed on the Illimani Glacier^61^ (located ~55 km from the Zongo Glacier); (c) a snowpack thickness of 2.6 meters based on snow cover accumulated from December 1999 to May 2000 at 5,150 meters asl^59^ (the albedo of the underlying ground did not contribute to the net result due to the snowpack thickness adopted); (d) BC concentrations of 73.4 ppb, 41.1 ppb and 29.25 ppb from our modeling results for September 2010, September 2007 and October 2010, respectively; and (e) dust concentrations of 10 and 100 ppm from previous measurements at Zongo Glacier^37^.
6. Energy flow available for snow melting (Qm) was calculated using the energy balance equation according to Sicart *et al*.^43^:
  2 $$Qm=R+H+LE+P$$
  where Qm is the energy used for melting (when positive) or freezing (when negative). R is the radiation balance; H and LE are the turbulent flows of sensible and latent heat, respectively; and P is the heat advected by precipitation. P remains very low over Zongo Glacier and was neglected. Therefore, we assumed that:
  3 $$Qm=R+H+LE$$
  Net radiation can be written as follows:
  4 $${\rm{R}}={\rm{S}}\downarrow -{\rm{S}}\uparrow +{\rm{L}}\downarrow -{\rm{L}}\uparrow $$
  where S ↓ is the incident shortwave radiation; S ↑ is the reflected shortwave radiation; and L ↓ and L ↑ correspond to incident and emitted longwave radiation, respectively. Since the former is equal to the amount of incident shortwave energy not reflected by the surface, net shortwave radiation (S ↓ - S ↑) can be rewritten by incorporating albedo into the equation. As albedo is a variable that describes the percentage of reflected energy, the term S ↓ - S ↑ in Eq. (4) can be written as follows:
  5 $${\rm{R}}={\rm{S}}\downarrow (1-{\rm{albedo}})+{\rm{L}}\downarrow -{\rm{L}}\uparrow $$
  Net longwave radiation can also be rewritten by considering emissions and the surface temperature according to Stefan-Boltzmann’s law. Thus, the term L ↓ - L ↑ can be substituted, resulting in the following net radiation equation:
  6 $${\rm{R}}={\rm{S}}\downarrow (1-{\rm{albedo}})+{\rm{L}}\downarrow -{{\rm{\varepsilon }}{\rm{\sigma }}{\rm{T}}}_{s}^{4}$$
  where ε is the longwave emission of ice, σ is Stefan-Boltzmann’s constant (σ = 5.67 10-8 W m^−2^ K^−4^), and Ts is the surface temperature. The ice emission, ε, is generally considered to be between 0.97 and 1.00. Ice was considered to be a complete emitter (ε = 1). This consideration is acceptable considering the accuracy of the longwave radiation measurements (approximately ± 10%)^43^. Turbulent heat flows were calculated using the bulk aerodynamic method, including the stability correction. The stability of the surface layer was described by the bulk Richardson number, Rib, which relates the relative effects of buoyancy to mechanical forces^62^:
  7 $${\rm{Rib}}=\frac{{\rm{g}}\frac{({\rm{T}}-{T}_{s})}{({\rm{z}}-{z}_{0{\rm{m}}})}}{T{(\frac{u}{{\rm{z}}-{{\rm{z}}}_{0{\rm{m}}}})}^{2}}=\frac{g\,({\rm{T}}-{\rm{Ts}})\,({\rm{z}}-{z}_{0{\rm{m}}})}{T{u}^{2}}$$
  where T and u are the air temperature (in K) and the horizontal wind speed (in m s^−1^), respectively, at the level of the measurement, z; g is the acceleration due to gravity (g = 9.81 m s^−2^); Ts is the surface temperature (in K); and z_0m_ is the surface roughness length for momentum (in m). Assuming that the local gradients of the mean horizontal wind speed, u; mean temperature, T; and mean specific humidity, q, are equal to finite differences between the measurement level and the surface, the turbulent flows are^62^:
  8 $$H={\rm{\rho }}\frac{{c}_{p}\,{k}^{2}u(T-{T}_{s})}{(\mathrm{ln}\,\frac{z}{{z}_{0m}})(\mathrm{ln}\,\frac{z}{{z}_{0T}})}({{\rm{\varphi }}}_{m}\,{{\rm{\varphi }}}_{H})-1$$
  9 $$LE={\rm{\rho }}\frac{{c}_{p}\,{k}^{2}u(T-{T}_{s})}{(\mathrm{ln}\,\frac{z}{{z}_{0m}})(\mathrm{ln}\,\frac{z}{{z}_{0T}})}({{\rm{\varphi }}}_{m}\,{{\rm{\varphi }}}_{H})-1$$
  where qs is the specific humidity of the surface (in g kg^−1^, where saturation is assumed), ρ is the air density, *C*_*p*_ is the specific heat capacity for air at a constant pressure (C_p_ = C_pd_ (1 + 0,84q), with C_pd_ = 1005 j kg^−1^ k^−1^), L_s_ is the latent heat of the sublimation of ice (L_s_ = 2.834 10^6^ j kg^−1^), and k is the von Karman constant (k = 0.4). The three roughness lengths are set equal to one another (z_0T_ = z_0q_ = z_0m_) and are used as calibration parameters for fitting the calculated sublimation. The surface roughness length in the Zongo Glacier varies from 1 to 5 mm^42^. The nondimensional stabilities of momentum (_*ϕm*_), heat (_*ϕH*_)), and moisture (ϕ_*v*_) are expressed in terms of Ri_b_^63,64^:
  Ri_b_ positive (stable)
  10 $${({{\rm{\varphi }}}_{m}{{\rm{\varphi }}}_{H})}^{-1}={({{\rm{\varphi }}}_{m}{{\rm{\varphi }}}_{v})}^{-1}={(1-5{\rm{Rib}})}^{2}$$
  Ri_b_ negative (unstable)
  11 $${({{\rm{\varphi }}}_{m}{{\rm{\varphi }}}_{H})}^{-1}={({{\rm{\varphi }}}_{m}{{\rm{\varphi }}}_{v})}^{-1}={(1-16{\rm{Rib}})}^{0.75}$$
  We analyzed the energy balance from 2005 to 2011. The impact of BC deposition was analyzed for critical years from 2007 to 2010. The meteorological data used to calculate the energy balance were obtained from AWS2, which was located on the glacier at 5,060 meters above sea level^43^.
7. The energy available for melting, taking into consideration the BC-albedo effect (Qm*), was calculated using Eq. (6) by incorporating an albedo reduction due to BC deposition.
8. A linear regression model was constructed based on the relationship between the energy balance (the energy available to melt) (Qm) and the annual mass balance measured within the ablation zone. The snow/ice mass loss due to BC was calculated using Qm* values generated from various BC concentrations.

### Modeling water discharge using meteorological and aerosol index data

To assess the relationship between water discharge on the Zongo Glacier and meteorological parameters/the Amazonian aerosol index, we applied a backward stepwise multiple regression analysis using SigmaPlot® software. The parameters used to predict water discharge were air temperature, humidity, precipitation, incident shortwave radiation, net longwave radiation (all meteorological parameters were measured by the automatic weather station located on the Zongo glacier), and the Amazonian aerosol index (with a 3-day lag) (calculated for the Amazon Basin from MODIS satellite observations). The period analyzed ranged from 5 August 2010 to 18 September 2010.

## Supplementary information

Supplementary material
