# Supplementary material for: Amazonian Biomass Burning Enhances Tropical Andean Glaciers Melting
Source: Sci Rep. 2019 Nov 28;9:16914. doi: 10.1038/s41598-019-53284-1 (PMC6882791; doi:10.1038/s41598-019-53284-1)
Supplement: Supplementary file 1 — Supplementary material [file 41598_2019_53284_MOESM1_ESM.pdf]

AMAZONIAN BIOMASS BURNING ENHANCES TROPICAL ANDEAN GLACIER MELTING

Newton de Magalhães Neto*[1,2,3]; Heitor Evangelista[2,3]; Thomas Condom[4]; Antoine Rabatel[4] and [4]Patrick Ginot

*Corresponding author (email address: newtonmagalhaesbio@hotmail.com)

[1] Laboratory of Geoprocessing and environmental studies, Institute of Geography, Rio de Janeiro State University – Rio de Janeiro, Brazil

[2]Geochemestry PHD program, Federal Fluminense University - Niteroi – Rio de Janeiro, Brazil

[3] Laboratory of Radioecology and Global Change, Institute of Biology Roberto Alcantara Gomes, Rio de Janeiro State University – Rio de Janeiro, Brazil

[4]Univ. Grenoble Alpes, CNRS, IRD, Grenoble-INP, Institut des Géosciences de l’Environnement (IGE, UMR 5001), F-38000 Grenoble, France

Supplementary Figures

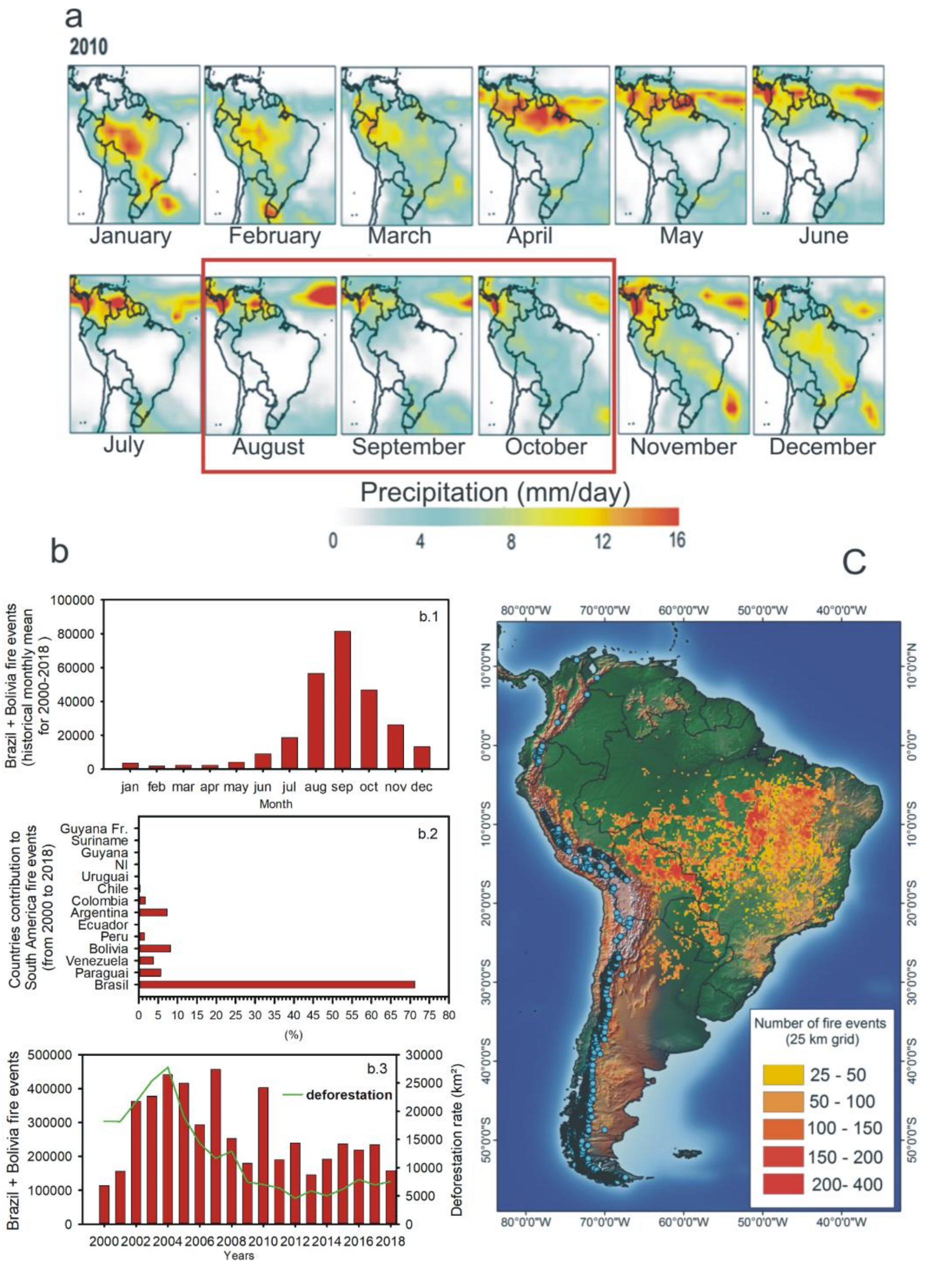

Supplementary Figure 1. Fire season in the Brazilian, and Bolivian Amazon Basins occurs during the transition period between the dry and wet seasons (from August to October). (a) Monthly mean precipitation maps for the year 2010 (data from Global Precipitation Climatology Project - GPCP[1]). (b) b.1 Historical monthly means of fire events in Brazil and Bolivia. b.2 Country contributions for the total number of fire events in South America. b.3 Mean interannual variability of fire events in Brazil and Bolivia (data from the INPE fire database[2]) and deforestation rate in Brazil (data from INPE deforestation monitoring program). (c) The spatial distribution of fire events during the 2010 fire season (25 km grid) and glaciers locations (blue circles) (data from the Randolph Glacier Inventory database[3]). Approximately 70% of fire events occurred in Brazil, although fire events in Bolivia were also significant and were closest to the Andean Glaciers.

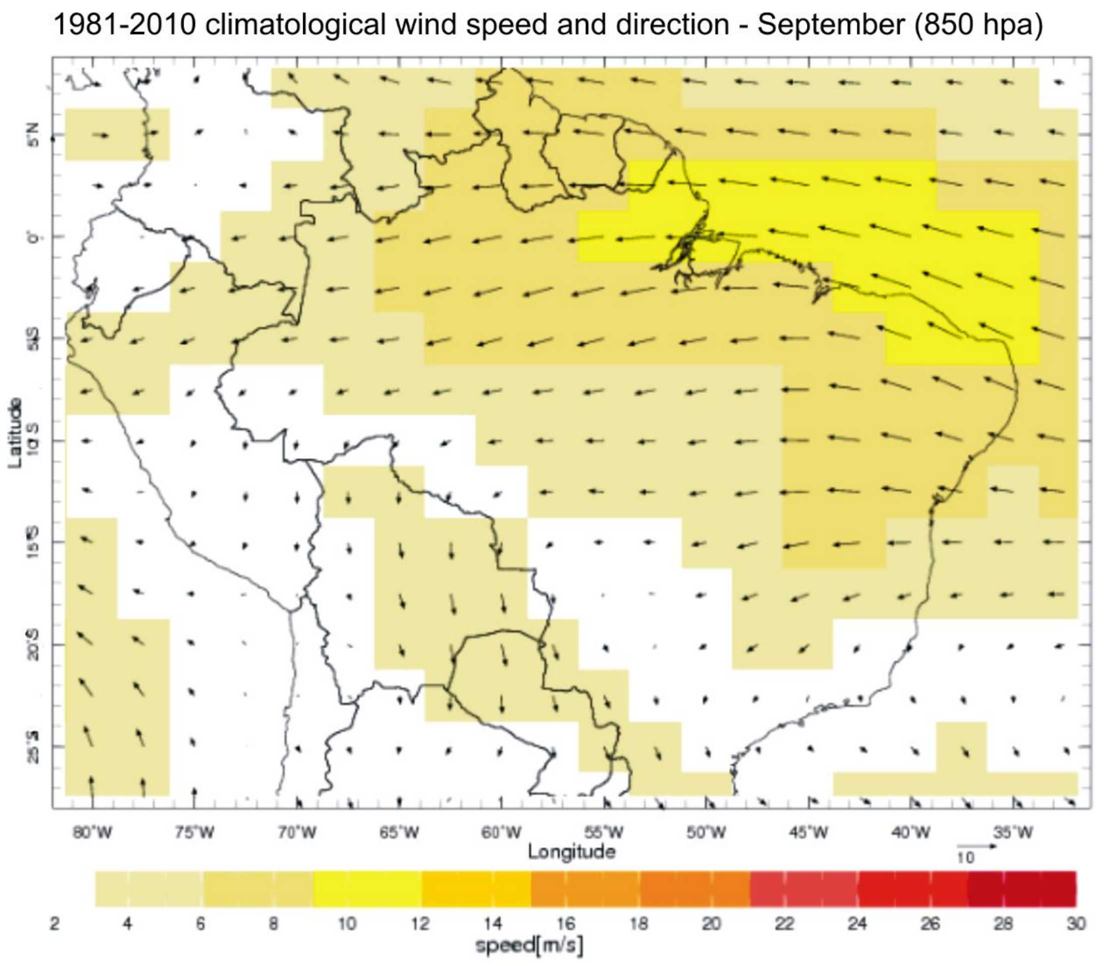

Supplementary Figure 2. Monthly climatology of September average winds using the 1981-2010 base period. The data corresponds to NCEP-NCAR Reanalysis monthly zonal and meridional winds at 850 hpa on a 2.5° lat/lon grid.

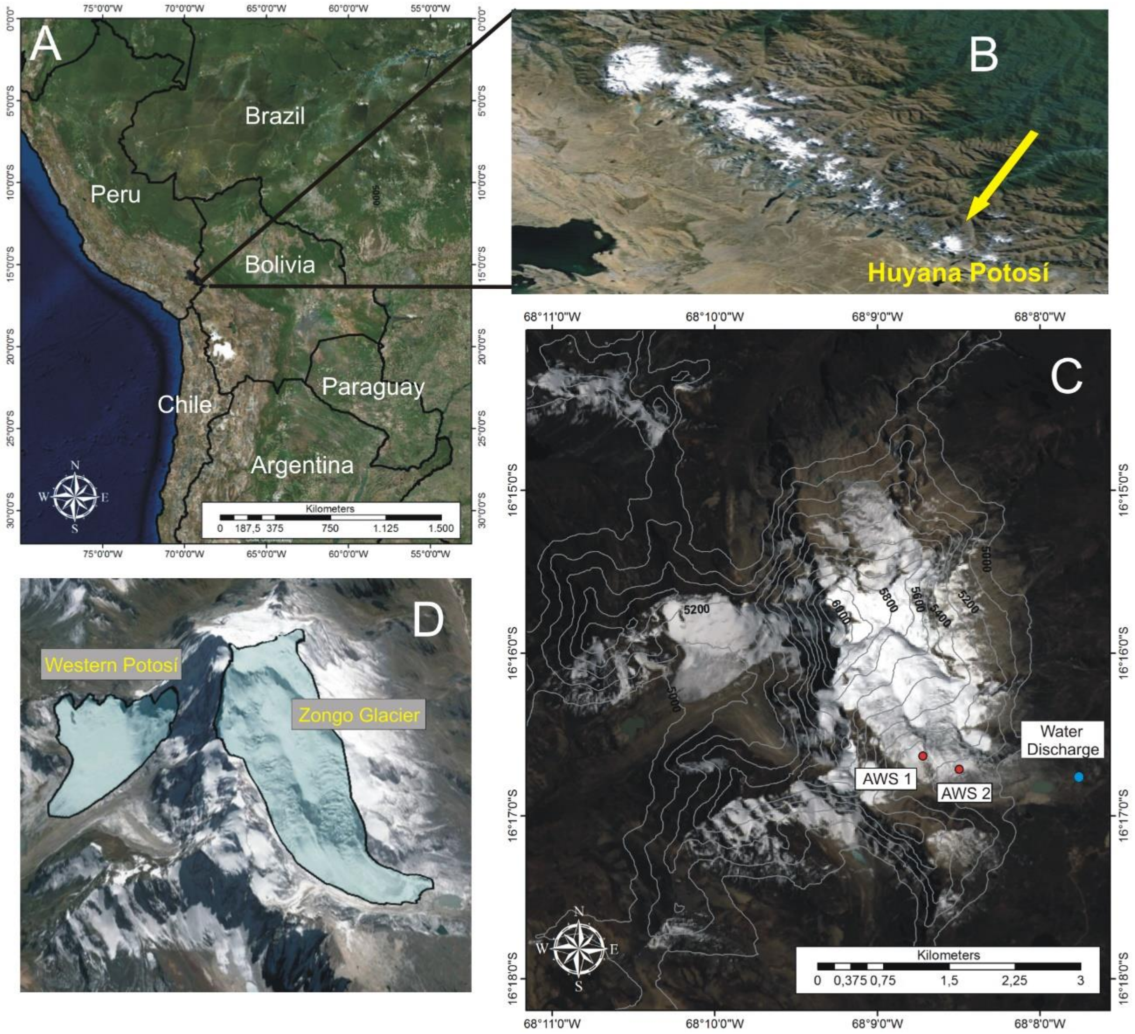

Supplementary Figure 3. Location of the Zongo Glacier. (a) The Zongo Glacier is located in the Huayna Potosi Massif (16°15′S, 68°10′W, Cordillera Real, Bolivia) on the western margin of the Amazon Basin and on the eastern margin of the Altiplano. The image is a MODIS 16-day composition MOD44C product from http://www.landcover.org./. (b) The Cordillera Real Mountains and the location of the Huyana Potosí Massif (Image: MODIS 16-day composition MOD44C product from http://www.landcover.org./). (c) The location of the two automatic weather stations on Zongo Glacier (red dots) and the water discharge measurements (blue dot). (d) Delimitation of the Zongo Glacier. The images in (c) and (d) are True color band composition (4-red, 3-green, 2-blue) from Landsat 8/OLI sensor collected in September 16, 2015 and May 29, 2016, respectively (images source: USGS earthexplorer, https://earthexplorer.usgs.gov/) (band composition were made using ArcGIS® 10.2).

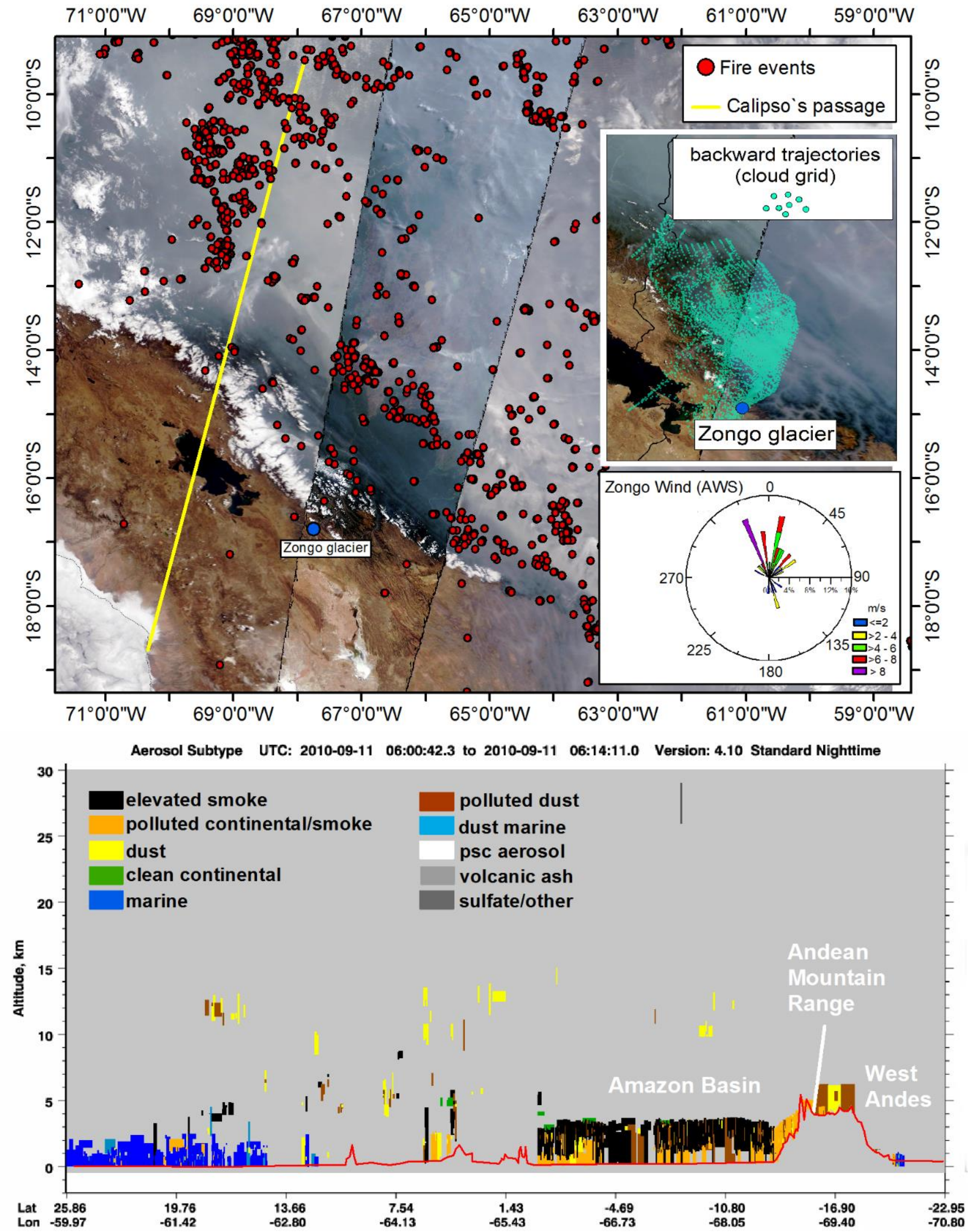

Supplementary Figure 4. CALIPSO-CALIOP smoke transport analysis. (a) daily MODIS true color band composition (1-red, 4-green, 3-blue) from AQUA satellite, captured on 09/11/2010 (NASA Worldview application, link to the images: https://worldview.earthdata.nasa.gov/09_11_2010); Hysplit backward trajectories from the Zongo Glacier, fire events location and wind speed and direction at the Zongo glacier. (b) Vertical profile of aerosols over the Andean mountain rage during the fire event on 09/11/2010.

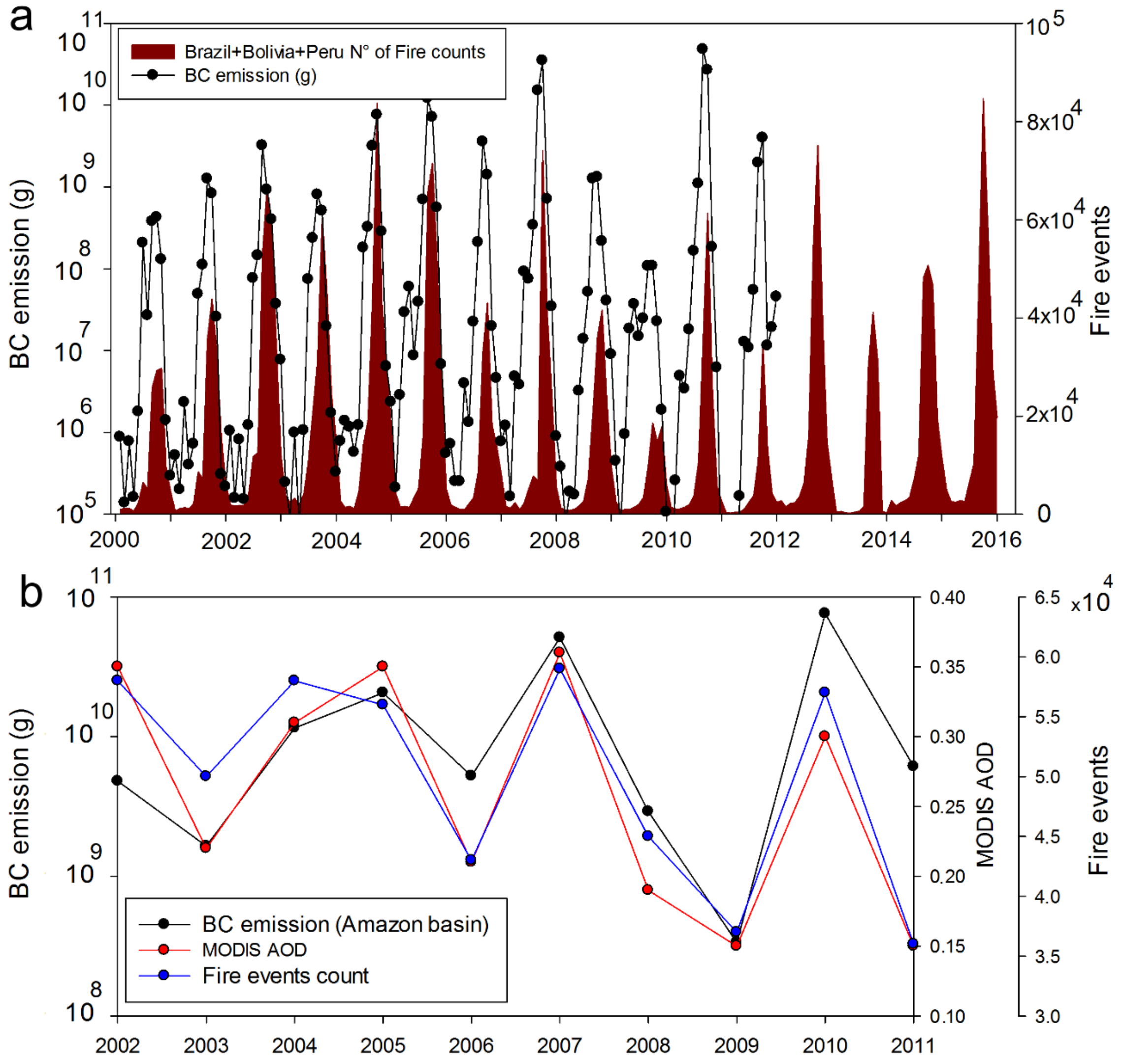

Supplementary Figure 5. Number of Fire events and black carbon emission within the Amazon Basin (a) Modeled monthly emissions of BC within the Amazon Basin (data from the global fire emission database - GFEDBv4[4]) and fire events occurring in Brazil, Bolivia, and Peru (data from the INPE fire database[2]) from 2000 to 2015. (b) Annual modeled BC emissions within the Amazon Basin (data from the global fire emission database - GFEDBv4[4]); MODIS aerosol optical depth (AOD); and the number of fire events within the Amazon Basin between 2002 and 2011 (data from the INPE fire database[2]).

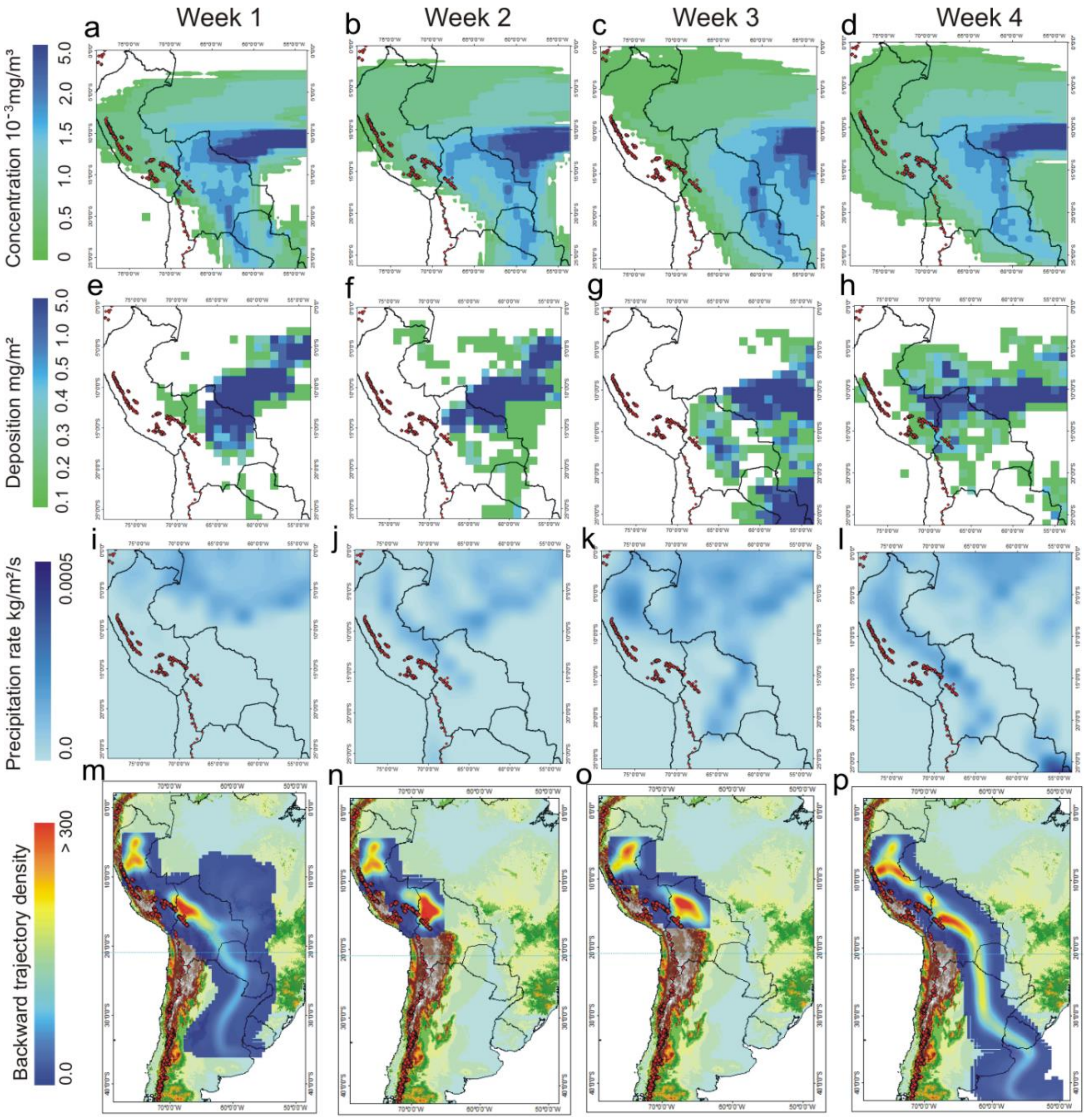

Supplementary Figure 6. Hysplit Model results for September 2007. (a, b, c, and d) the atmospheric black carbon concentration; (e, f, g, and h) the black carbon deposition; (i, j, h and l) the precipitation rate (data from NCEP/NCAR Reanalysis[4]); and (m, n, o, and p) the backward trajectories. Red dots represent glacier locations (data from the Randolph Glacier Inventory database[3]).

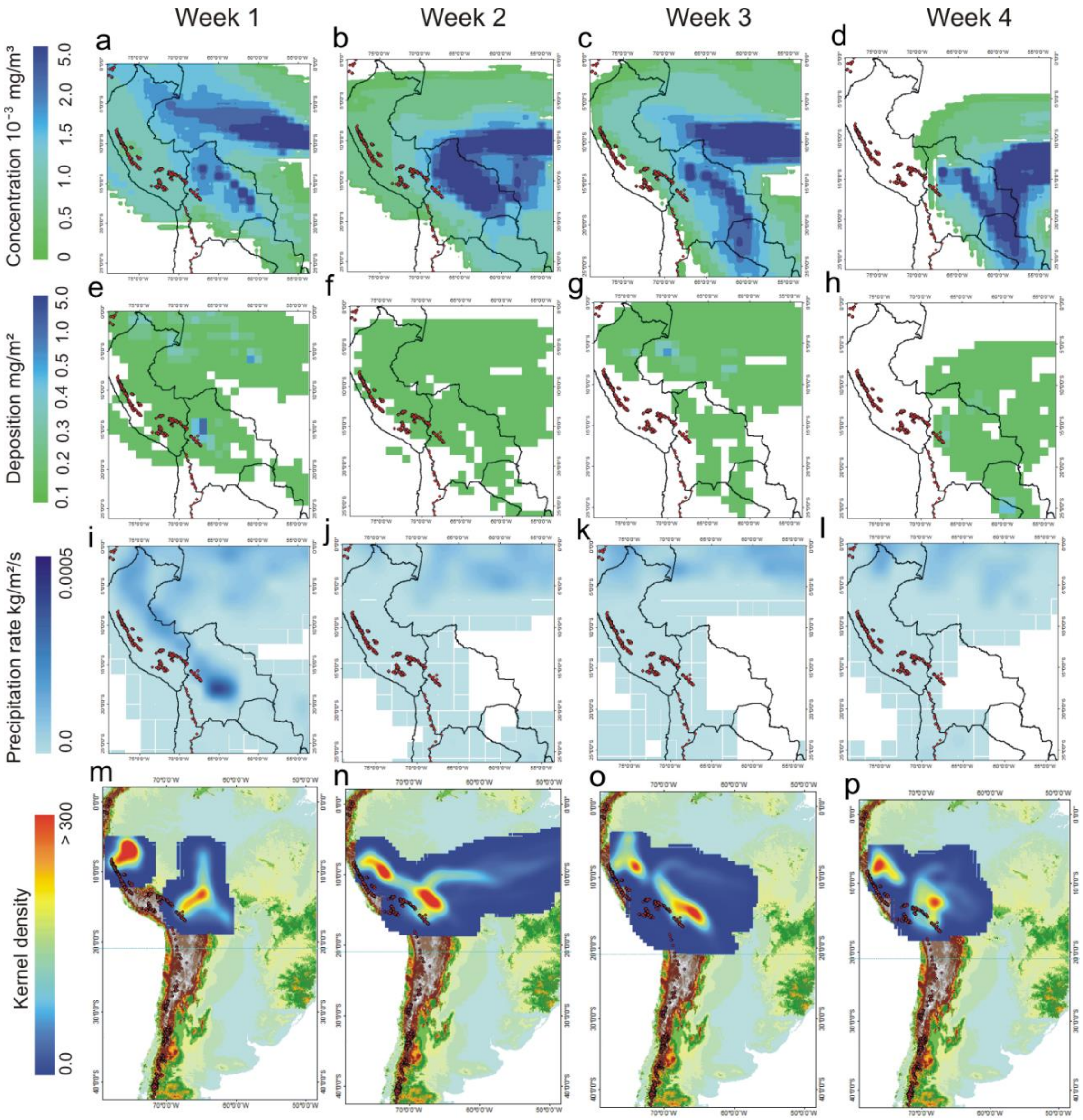

Supplementary Figure 7. Hysplit Model results for September 2010. (a, b, c, and d) the atmospheric black carbon concentration; (e, f, g, and h) the black carbon deposition; (i, j, h and l) the precipitation rate (data from NCEP/NCAR Reanalysis[4]); and (m, n, o, and p) the backward trajectories. Red dots represent glacier locations (data from the Randolph Glacier Inventory database[3]).

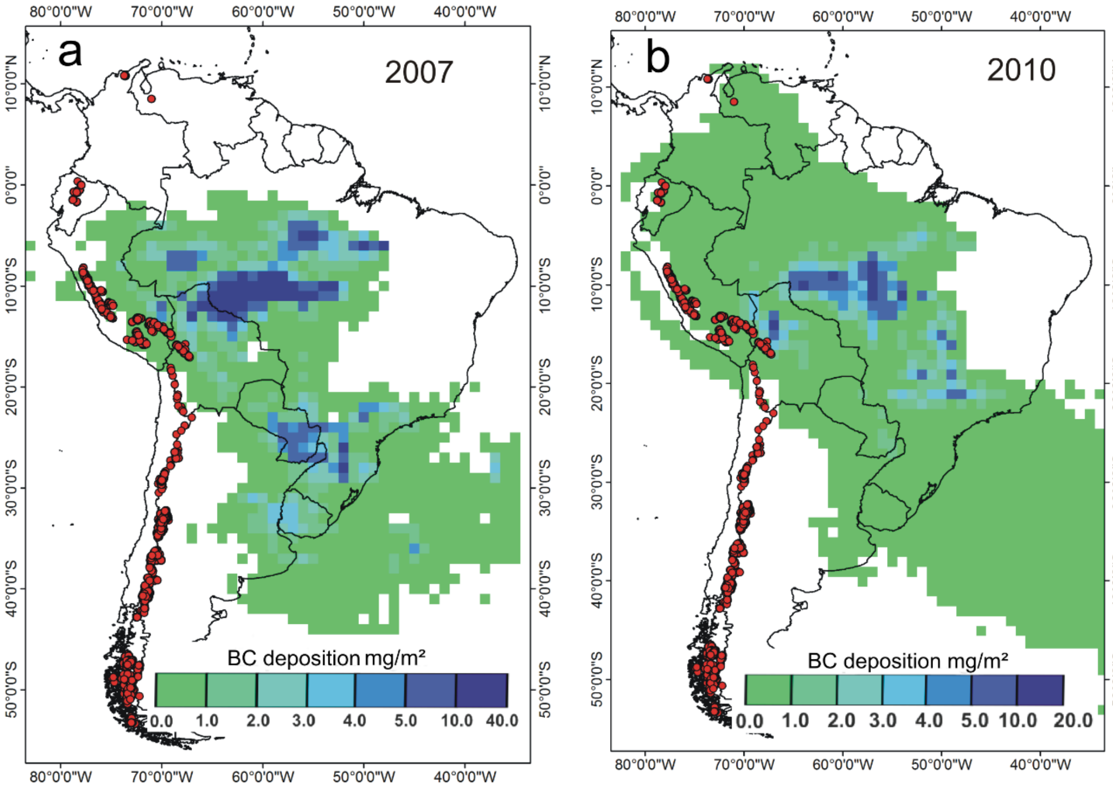

Supplementary Figure 8. Modeled black carbon deposition in South America during the 2007 and 2010 fire seasons. Hysplit/NOAA model output for total aerosol BC deposition during the fire season (August-September-October) of (a) 2007 and (b) 2010. The model was run on a weekly time scale for the 2007 and 2010 fire seasons. The red dots are glacier locations.

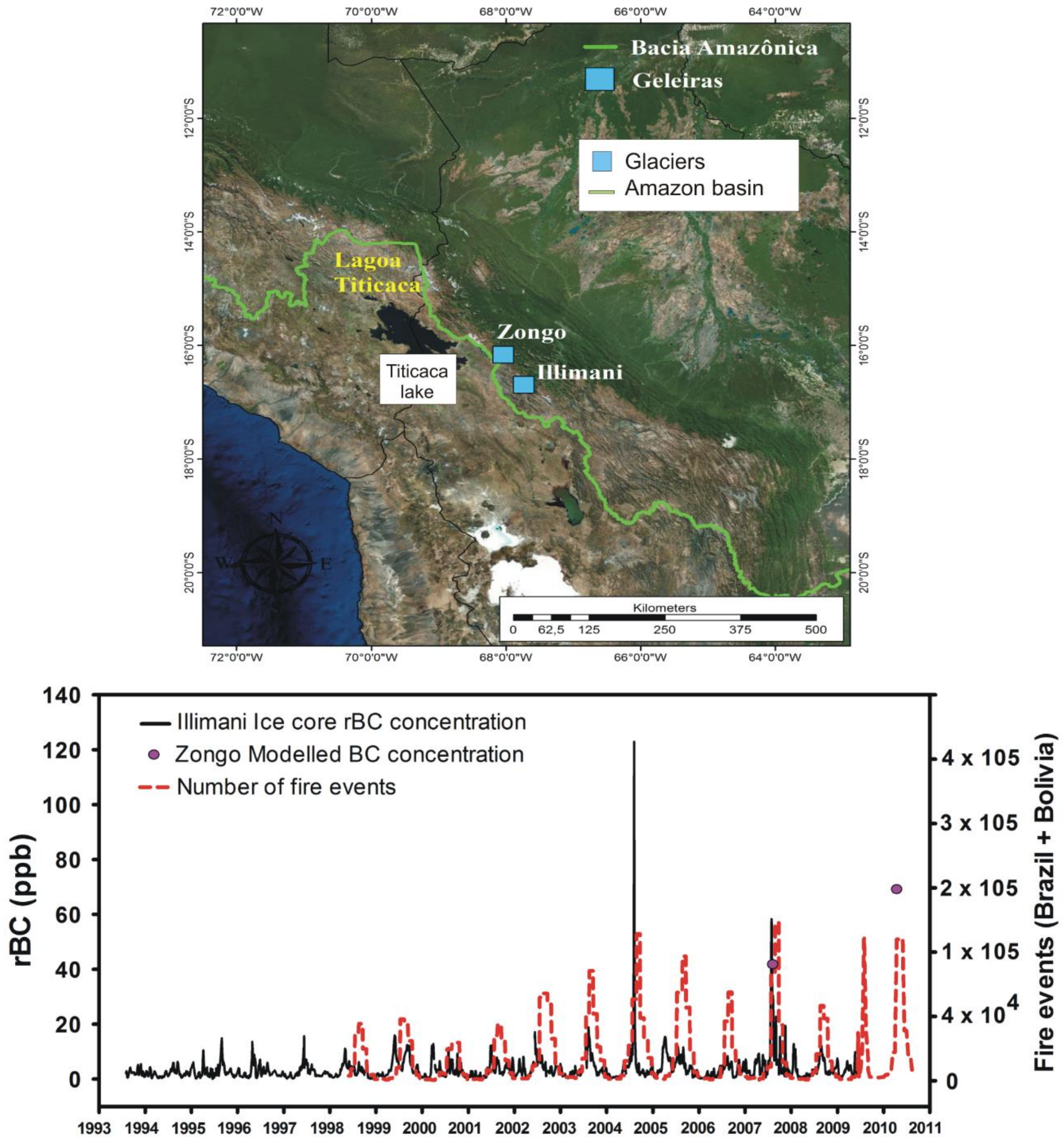

Supplementary Figure 9. Modeled and measured black carbon concentration (a) The Illimani and Zongo Glacier locations (The image is a MODIS 16-day composition MOD44C product from http://www.landcover.org./). (b) rBC concentration from an ice core collected at the Illimani Glacier spanning 1993-2009 (black line) and modeled BC concentration at the Zongo Glacier for 2007 and 2010 (purple circle). Illimani ice core was drilled in the summer of 2009 and ice layers were dated at LGGE (Laboratoire de Glaciologie et Geophysique de l'Environnement) using the seasonal signal of $\delta^{18}O$ varying from -27 to -10 pmil. Black carbon data, expressed in rBC, was measured in replicas of these samples using a single-particle soot photometer (SP2, Droplet Measurement Technologies) coupled to a nebulizer. The red dotted line shows fire event counts within the Amazon Basin (data from the INPE fire database[2]).

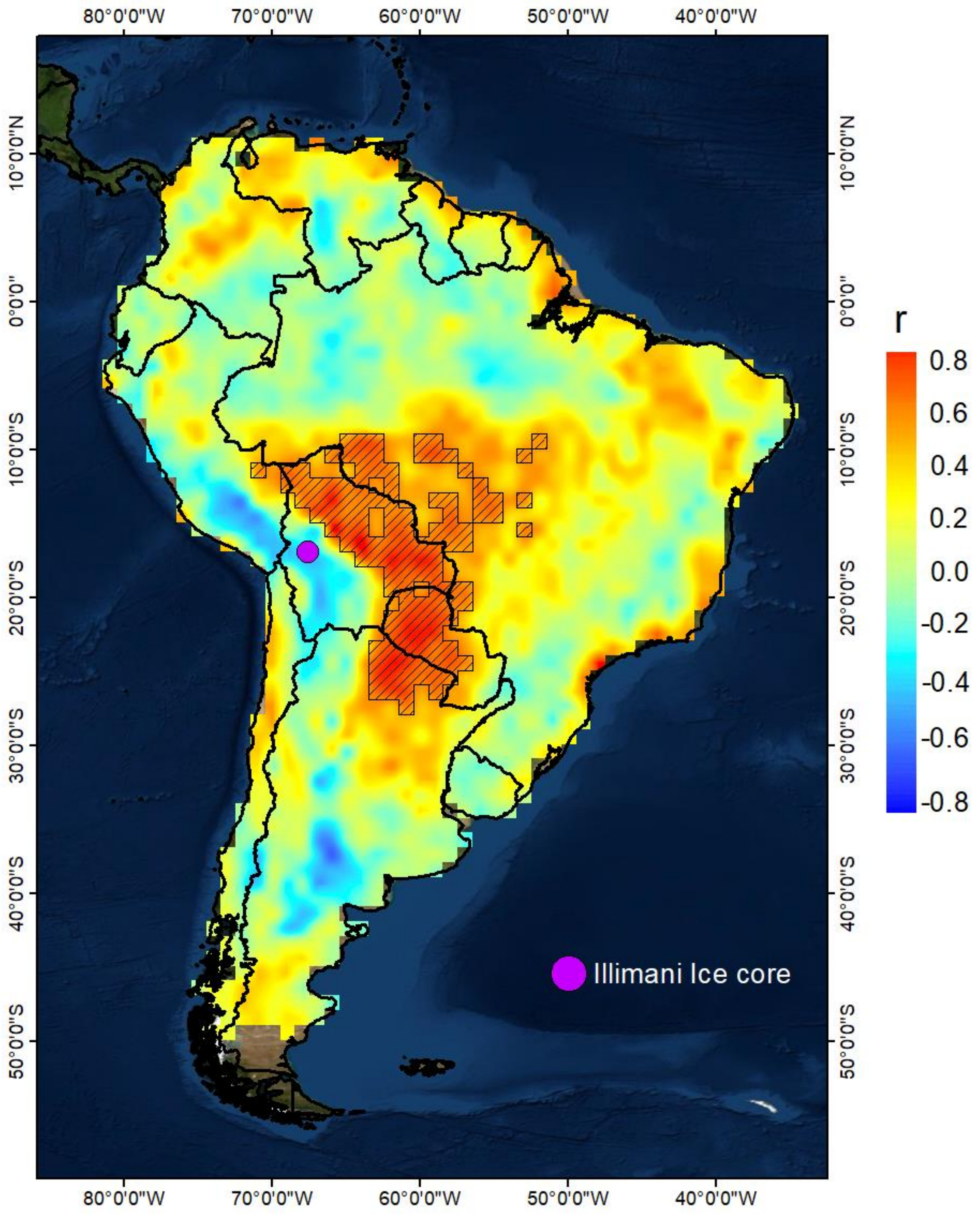

Supplementary Figure 10. Spatial correlation over South America between the Illimani rBC record and Aerosol Index for the 1994-2009. Hatching indicates statistically significance correlation (p<0.05) between Illimani rBC and aerosol index. Data are annual means.

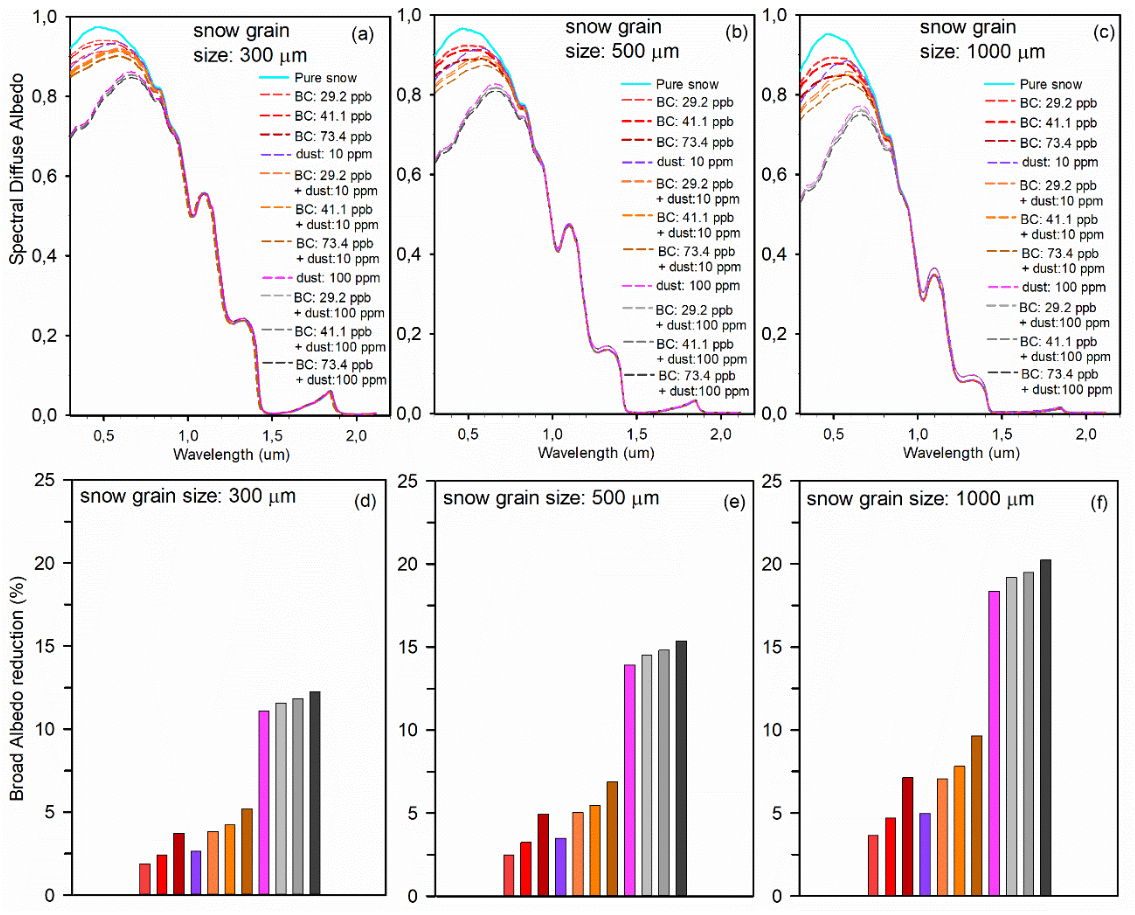

Supplementary Figure 11. Changes within the snow albedo of the Zongo Glacier simulated by the SNICAR model. (a), (b) and (c) for the visible-infrared spectrum at a 0.01 μm interval. (d), (e) and (f) The broadband albedo reduction for the shortwave spectrum. The broadband albedo is a weighted average of the albedo from all modeled spectral shortwave bands (from 0.3 - 5.0 μm), where the weight is the fraction of surface-incident spectral irradiance within each band.

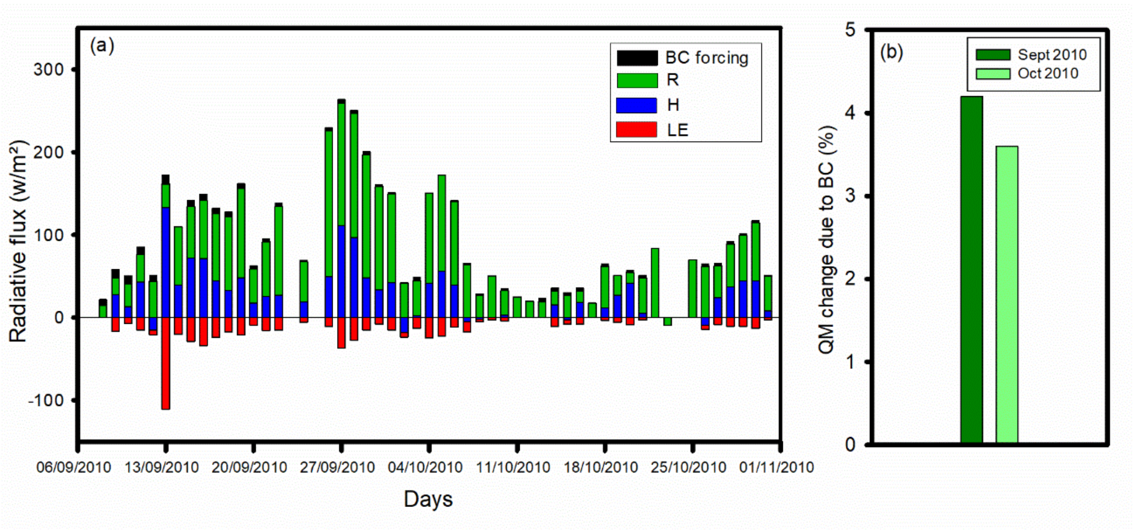

Figure 12. Contribution of the BC-albedo effect on the energy flux at the glacier's surface. (a) Daily values of the different energy balance terms (R, H, and LE) and the force due to BC on the Zongo Glacier during the fire season within the Amazon Basin (8 September to 31 October 2010). When the sum is positive, the total energy flux is first used to increase the temperature of the snow layer to the melting point when the surface temperature is negative, and then the energy is used to melt snow/ice. (b) The increase in the energy balance due to BC. Data used were from Zongo's AWS (on the glacier).

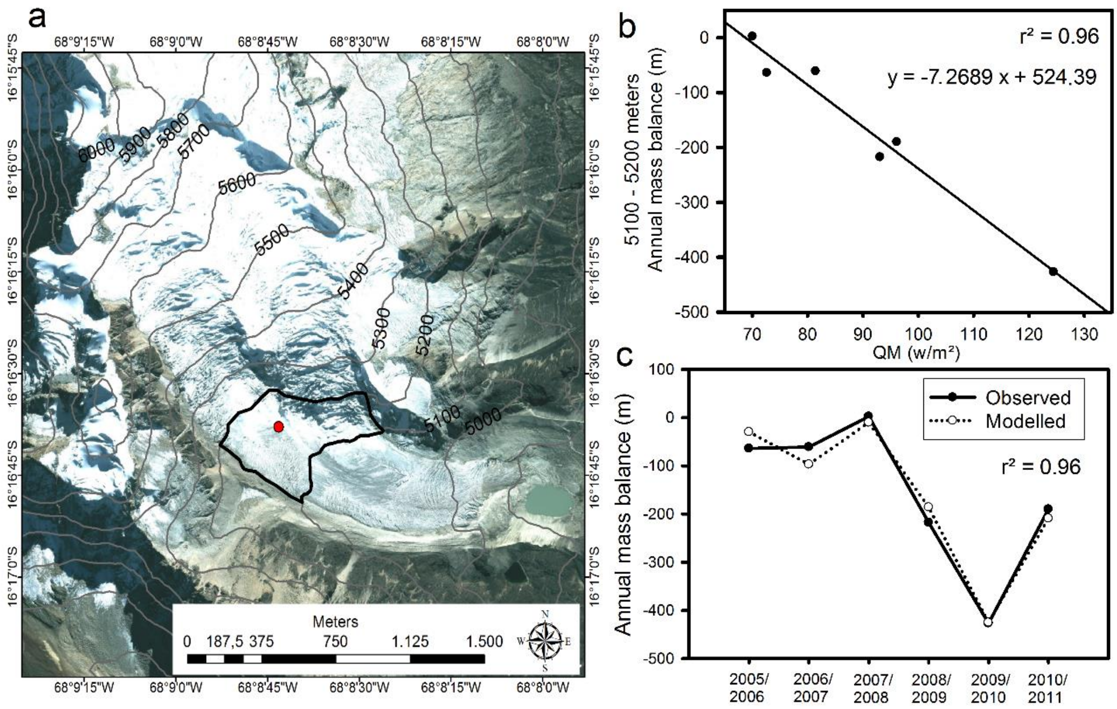

Supplementary Figure 13. Mass balance model based on energy balance. (a) The meteorological station used in the model (red dot) is located at an altitude interval between 5,100 and 5,200 meters (black line). The image used is from ArcGIS® 10.2 basemap (Source: Esri, DigitalGlobe, GeoEye, Earthstar Geographics, CNES/Airbus DS, USDA, USGS, AEX, Getmapping, Aerogrid, IGN, IGP, swisstopo, and the GIS User Community). (b) The linear regression model between the mass balance and the energy balance show statistical significance (Statistical significances were based upon a Student t test, $P < 0.001$, $n = 6$, $r^2 = 0.96$). (c) Observed and modeled mass balance.

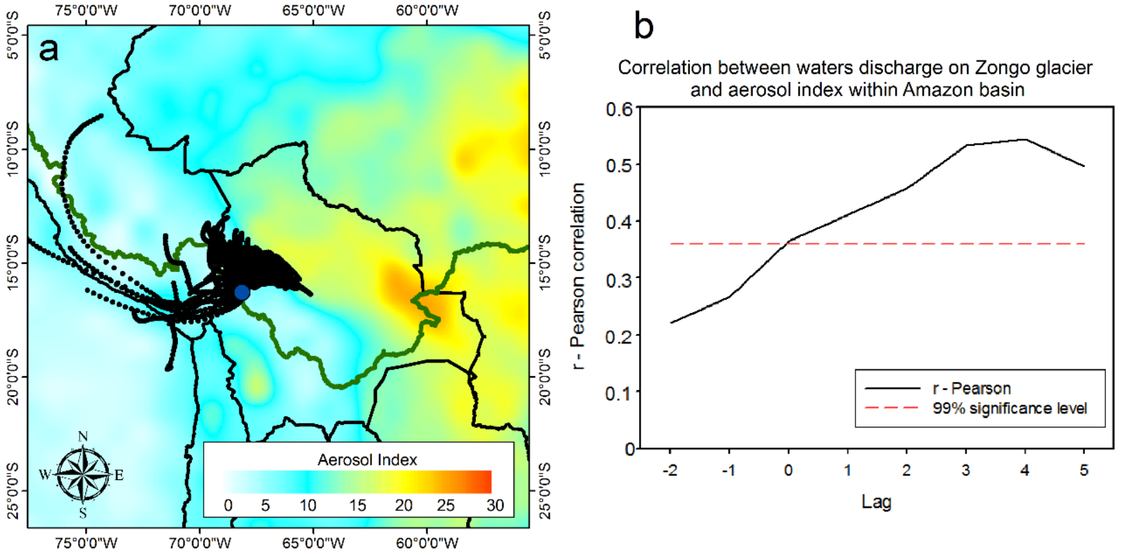

Supplementary Figure 14. peak water discharge from 5 August 2010 to 18 September 2010 displayed a statistically significant correlation with the three-day lag, daily MODIS Aerosol Index for the Amazon Basin. (a) Three-day backwards trajectories from the Zongo Glacier (trajectories were obtained using the Hysplit/NOAA Model) and the averaged Aerosol Index for September 2010. (b) The correlation for different lag times between water discharge in the Zongo Glacier and the Aerosol Index for the Amazon Basin.

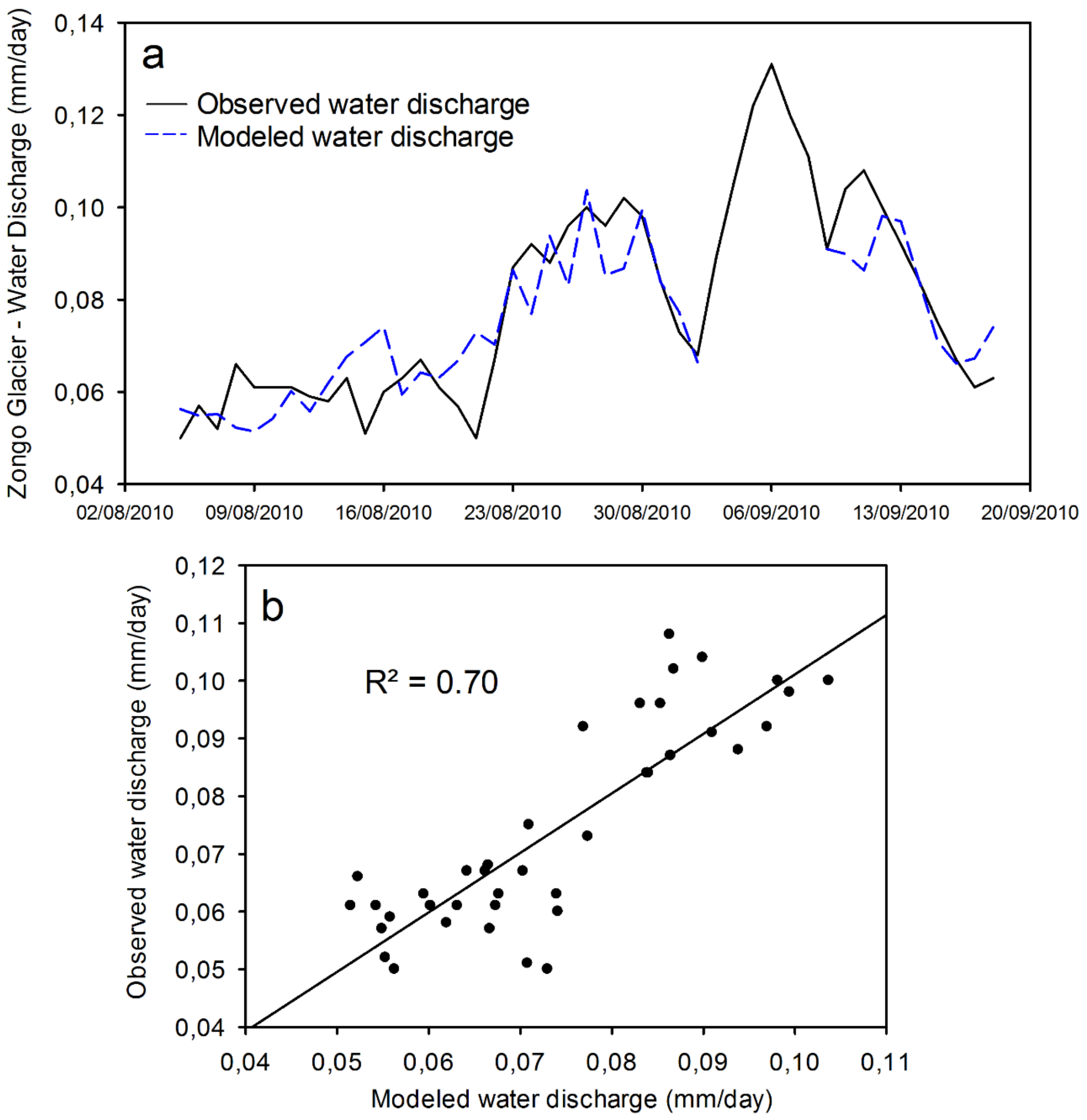

Supplementary Figure 15. A backward stepwise multiple regression analysis was performed in order to assess the relationship between water discharge on the Zongo Glacier and meteorological parameters/Amazonian Aerosol Index. The parameters tested during the modeling process to predict water discharge were: air temperature, humidity, precipitation, incident short wave radiation, net long wave radiation (all meteorological parameters measured by the automatic weather station located on the Zongo Glacier), and the Amazonian Aerosol Index (with 3 days lag) (calculated for the Amazon Basin from MODIS satellite observations). Among predictors parameters, the Aerosol Index and the radiation flux (incident shortwave radiation and net long-wave radiation) were statistically significant predictors of water discharge, explaining 70% of the variance (t-tests for partial coefficients, $P < 0.01$; for the full model, $F_{266} = 27.74$, $P < 0.001$; $r^2 = 0.70$). Our modeled discharge was based on the multi linear regression of these parameters, resulting on the following equation: Water discharge = 0.0542 + (0.00166 * AI (3 days lag)) + (0.000111 * Incident short wave radiation) + (0.000277 * Net long wave radiation). (a) A time series of observed and modeled water discharge on the Zongo Glacier. (b) A linear regression between observed and modeled water discharge on the Zongo Glacier.

Supplementary Methods Figures

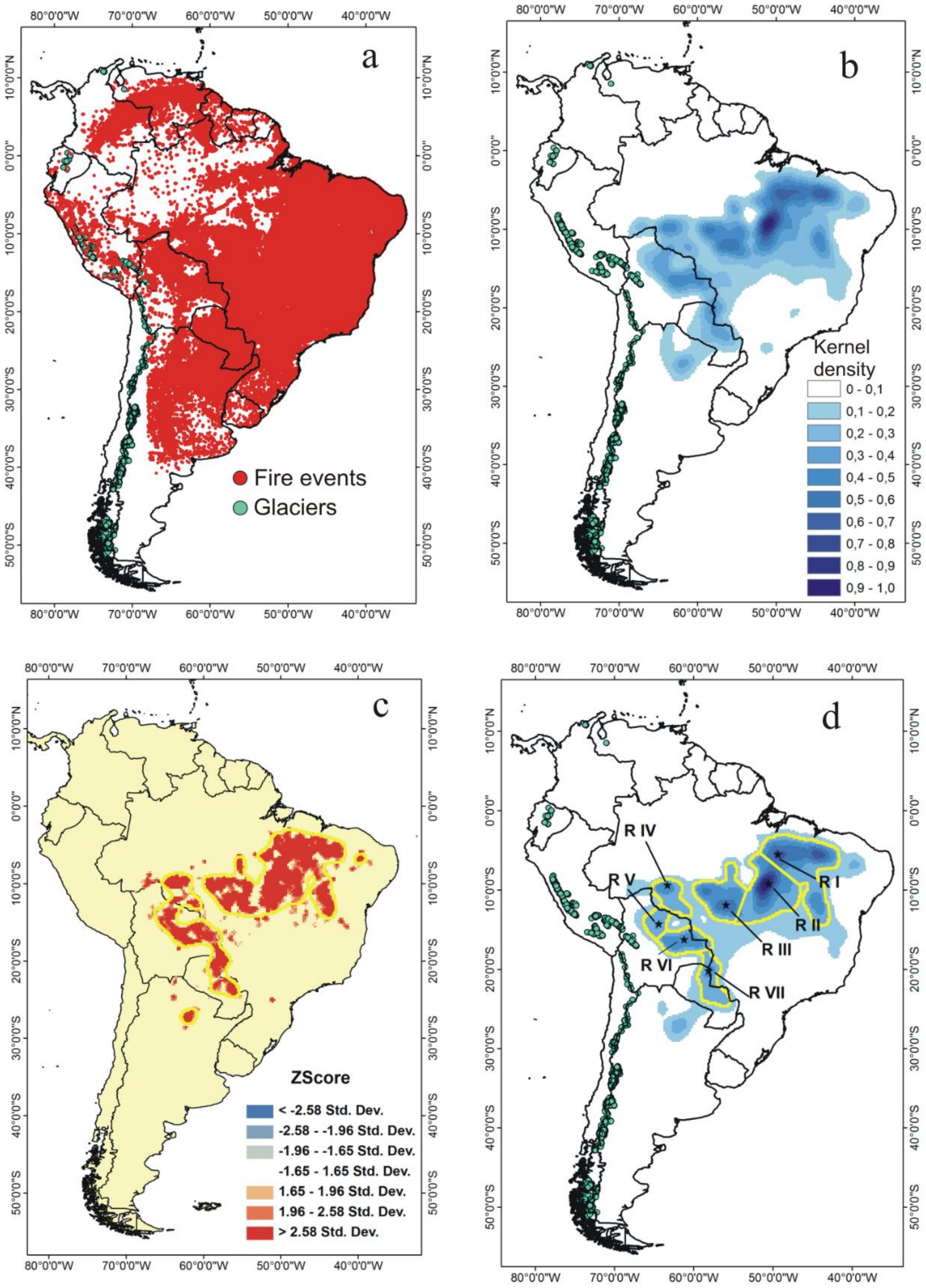

Supplementary Methods Figure 1. Determination of BC emission source regions. (a) Fire events from 2000 to 2016 (data from INPE fire database[2]). (b) The result of a kernel density analysis for the entire period. (c) The result of a Gi * analysis (statistically significant regions with a high density of fire events are shown in red and overlapped with results from the kernel analysis in yellow). (d) The seven emission regions used in the Hysplit model.

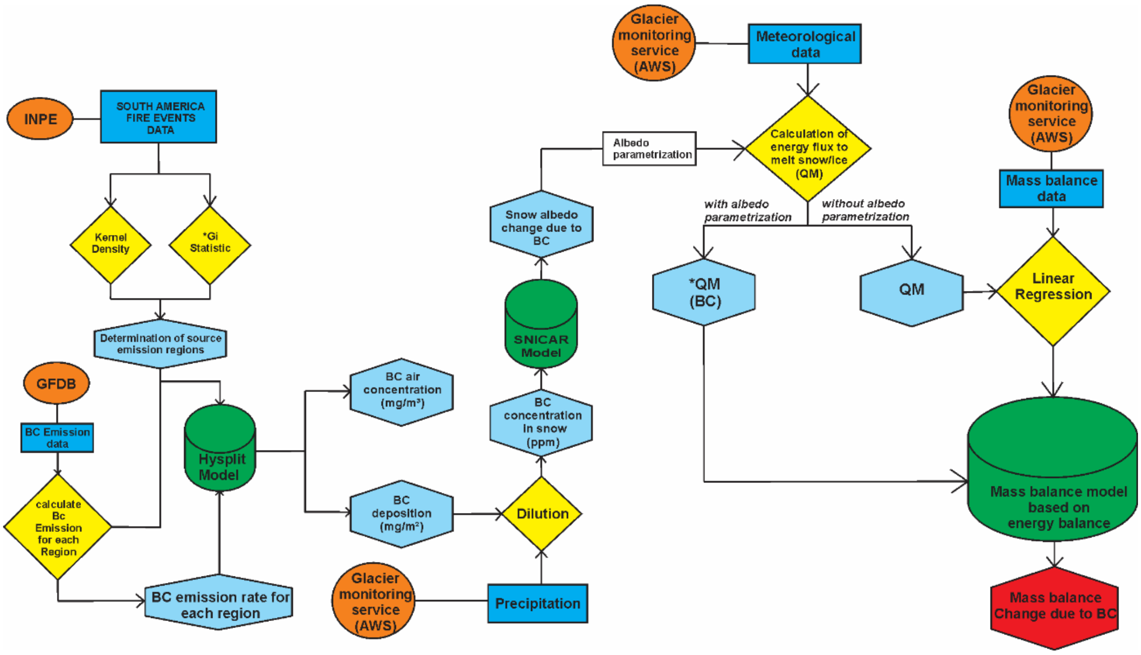

Supplementary Methods Figure 2. Methodological framework. The methodological framework used for the study of the impact of black carbon on the Zongo Glacier.

References

1. Adler, R.F., G.J. Huffman, A. Chang, R. Ferraro, P. Xie, J. Janowiak, B. Rudolf, U. Schneider, S. Curtis, D. Bolvin, A. Gruber, J. Susskind, and P. Arkin, 2003: The Version 2 Global Precipitation Climatology Project (GPCP) Monthly Precipitation Analysis (1979-Present). J. Hydrometeor., 4,1147-1167.

2. INPE - Instituto Nacional de Pesquisas Espaciais, 2018. Portal do Monitoramento de Queimadas e Incêndios (Wildfires monitoring program). http://www.inpe.br/queimadas.

3. RGI Consortium (2017). Randolph Glacier Inventory – A Dataset of Global Glacier Outlines: Version 6.0: Technical Report, Global Land Ice Measurements from Space, Colorado, USA.

4. Randerson, J.T., G.R. van der Werf, L. Giglio, G.J. Collatz, and P.S. Kasibhatla. 2018. Global Fire Emissions Database, Version 4, (GFEDv4). ORNL DAAC, Oak Ridge, Tennessee, USA. https://doi.org/10.3334/ORNLDAAC/1293
